# Impact of transgenerational host switch on gut bacterial assemblage in generalist pest, *Spodoptera littoralis* (Lepidoptera: Noctuidae)

**DOI:** 10.3389/fmicb.2023.1172601

**Published:** 2023-07-13

**Authors:** Amit Roy, Benjamin Houot, Sandeep Kushwaha, Peter Anderson

**Affiliations:** ^1^Faculty of Forestry and Wood Sciences, EXTEMIT-K and EVA.4.0 Unit, Czech University of Life Sciences, Suchdol, Czechia; ^2^Department of Plant Protection Biology, Swedish University of Agricultural Sciences, Alnarp, Sweden; ^3^Department of Bioinformatics, National Institute of Animal Biotechnology (NIAB), Hyderabad, India

**Keywords:** *Spodoptera*, holobiont, microbial assemblage, transgenerational host switch, host adaptation, 16S, larval gut bacteriome

## Abstract

Diet composition is vital in shaping gut microbial assemblage in many insects. Minimal knowledge is available about the influence of transgenerational diet transition on gut microbial community structure and function in polyphagous pests. This study investigated transgenerational diet-induced changes in *Spodoptera littoralis* larval gut bacteriome using 16S ribosomal sequencing. Our data revealed that 88% of bacterial populations in the *S. littoralis* larval gut comprise *Proteobacteria, Firmicutes, Actinobacteria*, and *Bacteroidetes*. The first diet transition experiment from an artificial diet (F0) to a plant diet (F1), cabbage and cotton, caused an alteration of bacterial communities in the *S. littoralis* larval gut. The second transgenerational diet switch, where F1 larvae feed on the same plant in the F2 generation, displayed a significant variation suggesting further restructuring of the microbial communities in the *Spodoptera* larval gut. F1 larvae were also challenged with the plant diet transition at the F2 generation (cabbage to cotton or cotton to cabbage). After feeding on different plant diets, the microbial assemblage of F2 larvae pointed to considerable differences from other F2 larvae that continued on the same diet. Our results showed that *S. littoralis* larval gut bacteriome responds rapidly and inexplicably to different diet changes. Further experiments must be conducted to determine the developmental and ecological consequences of such changes. Nevertheless, this study improves our perception of the impact of transgenerational diet switches on the resident gut bacteriome in *S. littoralis* larvae and could facilitate future research to understand the importance of symbiosis in lepidopteran generalists better.

**Importance:** Insect microbiota is recognized as a “hidden” player manipulating crucial traits in the insect. Out of >157,000 documented lepidopteran species, only <0.1% have been studied for bacterial symbionts. Hence, our current knowledge of bacterial symbiosis in lepidopteran insects is still restricted. We have limited information about the transfer of symbiotic bacteria between generations and the major drivers (i.e., ecological, morphological, and developmental) that shape lepidopteran gut microbiomes. This study elucidates the impact of transgenerational diet switches on the polyphagous pest (*S. littoralis*) larval gut bacteriome. The knowledge gathered from this study is necessary from a fundamental perspective to delineate the role of larval gut bacteriome in *Spodoptera* sp. holobiont and other polyphagous insects when switching between different host plants.

## 1. Introduction

The eco-evolutionary success of insects often depends on their combined ability to cope with environmental challenges as a holobiont (Douglas, 2015; Salem and Kaltenpoth, 2022). Microbial association influences various aspects of insect life, including digestion and detoxification for recalcitrant food; providing ecological immunity; protection against predators, pathogens, and parasites; providing essential amino acids, metabolic compounds, and nutrient supplements; mediating inter- and intra-specific communication; thermal stress; and controlling mating and reproductive success (Russell et al., 2014; Douglas, 2015; Arbuthnott et al., 2016; Wielkopolan and Obrepalska-Steplowska, 2016; Engl and Kaltenpoth, 2018; Gupta and Nair, 2020; Jing et al., 2020; Chakraborty and Roy, 2021; Chen et al., 2021; Singh et al., 2021). Recent reports also indicated insect microbe-mediated insecticide resistance (Xia et al., 2018; Bras et al., 2022; El Khoury et al., 2022; Hu et al., 2022; Zhao et al., 2022). Generalist herbivores, including lepidopteran insects, have acquired diverse mechanisms to handle challenges associated with feeding on several plant species or insecticide exposure, which include, but are not limited to, plasticity at gene expression and intricate symbiotic associations with microbes (Roy et al., 2016; Paniagua Voirol et al., 2018; Bras et al., 2022; Hu et al., 2022; Siddiqui et al., 2022; Zhang et al., 2022). Exploring core and differentially abundant gut microbiome communities in lepidopteran insect is a key to understanding how different host plants affect their development, behavior, adaptation, and preference (Paniagua Voirol et al., 2018; Zhang et al., 2022). The core microbial communities support conserved biological processes, and transient bacteriomes provide quick and plastic metabolic competencies against extrinsic disturbances or host switches (Otani et al., 2014; Jones et al., 2019; Chakraborty et al., 2020; Näsvall et al., 2021; Oliveira et al., 2022). Host feeding is a crucial factor underlying the gut microbial assemblage (Gayatri Priya et al., 2012; Jones et al., 2019; Lv et al., 2021; Malacrinò, 2022). However, some studies demonstrated no apparent contribution of symbiotic microbes to survival, weight gain, and developmental time in lepidopteran larvae (Appel, 2017), suggesting a failure to establish obligatory associations with free-living microbes. This may be due to a lack of specialized structures in the gut to hold microbes in or unfavorable alkaline environment or a high discrepancy in the microbial community associated with host plants making the selection and establishment of true symbionts evolutionarily unfavorable (Dow, 1984; Gayatri Priya et al., 2012; Staudacher et al., 2016; Hammer et al., 2017; Mazumdar et al., 2021). However, many other studies consistently documented the colonization of different bacterial groups in the gut of lepidopteran larvae, suggesting the prerequisites for further studies (Shao et al., 2014; Teh et al., 2016; Mason et al., 2020; Xia et al., 2020; Chen et al., 2022).

The polyphagous agricultural pest *S. littoralis* (Lepidoptera: Noctuidae) has been recorded to have more than 100 host plants and can reduce crop yield by up to 75% (Gaden et al., 2010; Douglas, 2015). It is present in Africa, Southern Europe, Iran, and Arabian Peninsula. In its agroecological environment, the availability of different cultivated crops changes throughout the year, which leads to host shifts for *S. littoralis* populations over the season. Female host plant choice decisions are based on an innate preference hierarchy and on larval and adult experience (Thöming et al., 2013; Proffit et al., 2015), where host plant quality as food is an essential factor (Lhomme et al., 2018). In addition, we have also found plant-dependent transgenerational effects on larval development, where the parental experience of a plant increases offspring performance (Rösvik et al., 2020). Matching parental and offspring host plants resulted in higher offspring weight than mismatching plants between the generations for one host plant but not for another host plant. Thus, host plant shifts and both inter- and transgenerational plasticity triggered by host plant feeding occur in *S. littoralis*, but the role of the larval gut microbiome in these processes is still largely unknown.

As host plant shifts are common in natural populations of *S. littoralis*, it can be ecologically beneficial for pest insects to maintain symbiotic relations with multiple microbes such as archaea, bacteria, fungi, and viruses (Gurung et al., 2019). Bacterial species are predominantly present in the digestive tract and act as crucial modulators in insect life cycle and behavior (Baumann et al., 2006). Several aspects of *Spodoptera spp*. microbiome were already investigated (Shao et al., 2014; Martínez-Solís et al., 2020; Ugwu et al., 2020, 2022; Xia et al., 2020; Mazumdar et al., 2021). For instance, a strategic microbiome-based investigation was performed to reveal bacterial species in *S. littoralis* egg, early and late instar larvae, pupa, and adults (Chen et al., 2016). 16S metagenome sequencing results showed overall low microbial diversity in the egg. However, substantially higher microbial diversity and composition shifts across the developmental stages of *S. littoralis* were documented. Proteobacteria is one of the predominant phyla in eggs, whereas a trend of phyla shift was observed from early instar larvae to the pupal stage. At the early instar larvae, the abundance of Firmicutes increased and became the predominant bacterial species at the late instar larval stage. Firmicutes were also dominated at the pupal stage. Interestingly, Proteobacteria regained dominance in adult males, whereas adult female microbiota consists of both Proteobacteria and Firmicutes. Overall, *Proteobacteria* (*Pantoea, Acinetobacter, Ralstonia*, and *Citrobacter*), *Firmicutes* (*Enterococcus* and *Clostridium*), and *Actinobacteria* have highly represented phyla within *S. littoralis* life cycle (Chen et al., 2016). In another independent study on *S. littoralis* larvae, *Pantoea, Citrobacter*, and *Clostridium* were dominant genera of early instar larvae as core functional populations (Shao et al., 2014). *Enterococcus* was also found to be metabolically active and consistent in the larval lifespan. *Enterococci* formed biofilm-like layers on the gut epithelium to establish a colonization resistance effect in the larval gut against potentially harmful microbes from outside (Shao et al., 2014). However, ecological relevance of such observations needs to be evaluated at the functional level.

Gut microbiome studies were also conducted on other *Spodoptera* species. The gut microbiota composition has been reported to be influenced by the environment and the diet in both *S. exigua and S. litura* (Martínez-Solís et al., 2020; Xia et al., 2020). The effects of the artificial and four plant diets on gut microbial community structure and diversity in *S. frugiperda* were also documented (Lv et al., 2021). *Proteobacteria, Firmicutes*, and *Bacteroidetes* dominated the gut microbial community, and the highest microbial abundance was reported on oilseed rape-fed larvae and the lowest microbial diversity on wild oat-fed larvae.

Limited studies have been conducted to evaluate the impact of the transgenerational diet switch on larval gut bacterial assemblage in generalist lepidopterans, including *Spodoptera* spp. Evaluating the larval gut bacterial assemblage upon transgenerational host switch for generalist pests, such as *S. littoralis*, that switch hosts between seasons will be intriguing. Such studies will facilitate a deeper understanding of the microbial contribution to host adaptation and also aid in identifying the conserved (core) microbial population in the gut. A profound understanding of the gut bacterial contribution to host adaptation can be used for formulating eco-friendly pest management practices, such as developing biochemical and biopesticides (i.e., RNAi-based products), that eliminate or disrupt insect symbiosis (Qadri et al., 2020; Joga et al., 2021; Zhang et al., 2022; Sandal et al., 2023), antimicrobial peptides (Carter et al., 2013), introducing a foreign microbe (Moran and Yun, 2015), incompatible insect technique (Atyame et al., 2016), paratransgenesis (Caragata and Walker, 2012), and pheromones alteration (Cardé and Minks, 1995). This study tries to elucidate the influence of matching and mismatching plant diets between generations on *S. littoralis* larval gut bacterial communities. Our results documented noteworthy changes in the *Spodoptera* larval gut bacterial assemblage upon each diet switch, enhancing our understanding of gut microbial associations influenced by transgenerational diet switch and demanding further experimental corroboration to understand its functional relevance.

## 2. Materials and methods

### 2.1. Plant and insect rearing

The plants were cultivated in 1.5 L pots in a commercial substrate (Krommull, Weibull Trädgard AB, Hammenhög, Sweden) for 5–6 weeks at 25 ± 2°C, 70 ± 5% relative humidity (R.H.) with artificial light (Osram Powerstar HQI-T. 400W/D. 16:8 h L.D. cycle) in the greenhouse until they were used for experiments. All plants were in a non-flowering stage when used. Based on earlier experiments, we selected two host plant species on which moths exhibited a similar performance but with different preferences: cotton (*Gossypium hirsutum*, DPL-90) and cabbage (*Brassica oleracea* v. capitata) (Thöming et al., 2013).

### 2.2. Feeding experience

**Figure 1:**
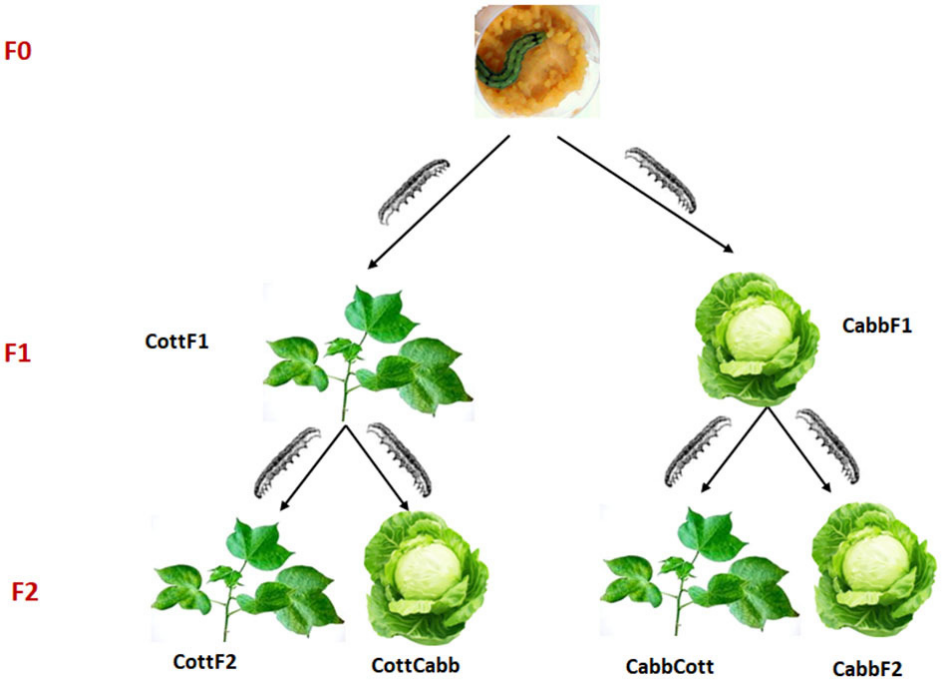
Experimental design to explore the effect of different diets and diet transition on *S. littoralis* gut microbiome.

The rearing moth strain of *S. littoralis* was collected from Egypt in 2008. Moreover, it was refreshed with new wild-collected moths yearly before the start of the experiments. The insect colony was reared on an artificial diet based on potatoes (Hinks and Byers, 1976; Roy et al., 2016) at 25 ± 2°C, RH: 65 ± 2%, and 17:7 h L:D cycle. At the pupal stage, males and females were separated until eclosion, and adults were used for mating when they reached 2 days. Five couples were selected and put into a cage containing the same host plant as the individual had experienced at the larval stage for mating. The transgenerational experiment involved rearing two consecutive generations of insects on two different host plants (cotton or cabbage) from the first instar until pupation. Adult males and females (10 each) from an artificial diet (AD) were collected and placed in a cylindric mating cage (height 15 cm. Ø 11 cm) with a cup that contained a cotton ball permeated with a 20% sucrose solution. F1 offspring generated were randomly divided into two groups; one group was fed cabbage (CabbF1), and the other group cotton (CottF1). First instar larvae from both plant diet-rearing groups were sampled for microbiome analysis. The offspring of each F1 generation (CottF1 and CabbF1) were again divided into two groups and reared either on the same host plant as their parents had experienced or switched to a new host plant (CottF2, CabbF2, CottCabb, and CabbCott) (Figure 1).

### 2.3. 16S amplicon sequencing and data analysis

At the eclosion, 200 larvae (4th instar) were dissected in 1× PBS, and larval guts were collected after the food bolus was removed using a soft brush. Finally, the guts were snap-frozen in liquid nitrogen and stored at −80°C for subsequent nucleic acid extraction, as described earlier (Roy et al., 2016). For each treatment, three biological replicates were collected. The total DNA was purified for each sample from 200 first instar larvae using DNeasy Blood and tissue kit (QIAGEN) following the manufacturer protocol. The extracted *Spodoptera* larval gut tissue DNA was quantified on Qubit 2.0 Fluorometer using a Qubit 2.0 High sensitivity dsDNA assay kit and electrophoresed on 1% agarose gel to evaluate the DNA integrity as per preoptimized lab protocol (Chakraborty et al., 2023). The concentration was set to 10 ng/μl of genomic DNA with a quantity of at least 200 ng in 20 μl. All the samples were stored at −20°C and shipped to LGC, Germany, for 16S amplicon sequencing [300 bp paired-end read (Illumina MiSeq V3) Bacteria 16S (515YF-926R)]. The sequencing reaction also included no template DNA as a negative control. Raw sequence data were demultiplexed and quality-filtered using QIIME (version 1.9.0). All the forward and reverse reads were merged using BBMerge tools, and merged read sequences were used to identify multiple operational taxonomic units (OTUs) at a similarity level of 97% through Mothur (1.35.1 software package) (Schloss et al., 2009) by using the 16S Mothur-Silva SEED r119. Putative species-level OTUs were annotated with NCBI BLAST+ 2.2.29 using BLAST+ parameters: E ≤ 0.1, percent identity ≥90%. OTU diversity analyses were performed using QIIME 1.9.0 (Caporaso et al., 2010), and alpha diversity, Chao1, Phylogenetic diversity, and Shannon and Simpson indices were estimated. The alpha diversity and relative abundance data were analyzed using Kruskal–Wallis one-way ANOVA by rank test (McKight and Najab, 2010). Venn diagrams and stack bars were graphed by R software (R CoreTeam, 2017). Principal coordinate analysis (PCoA) and permutational multivariate analysis (PERMANOVA) (Anderson, 2014) were performed based on the matrices of pairwise-weighted UniFrac distances and Bray–Curtis distances to identify the distribution pattern of different samples and to evaluate the significant differences between study groups, respectively. All the samples were clustered based on the abundances of specific taxa through Jackknifed UPGMA clustering using the weighted UniFrac metric (Lozupone et al., 2007). A dendrogram was drawn to show the distribution of samples at taxonomic composition levels (Top 10) using Chordial software. The OTU abundance was normalized across the samples, and extended error plots for the feeding experiments were generated using STAMP v.2.1.3 at 95% confidence intervals to explore the taxonomic and functional profiles of metagenomes (Parks et al., 2014). Differential abundance analyses (DAA) were performed based on normalized OTU counts using the DEseq2 package (Paulson et al., 2013). The metabolic profile of larval gut metagenomes was predicted using PICRUSt2 (Douglas et al., 2020) and the Kyoto Encyclopedia of Genes and Genomes (KEGG) database (Kanehisa et al., 2017) as per the PICRUSt2 tutorial (https://github.com/LangilleLab/microbiome_helper/wiki/PICRUSt2-tutorial). Furthermore, two different diet-fed larval samples were compared using Welch's *t*-test at 95% confidence intervals to explore significant functional differences between gut bacterial metabolic profiles (Delacre et al., 2017).

## 3. Result

The main aim of the study was to understand the transition of *S. littoralis* larval gut bacterial communities under the influence of the sequential change of three different diets (artificial diet, cabbage, and cotton). Larvae were switched from an artificial diet (F0) to a plant diet, either cabbage or cotton, in the F1 generation. In the F2 generation, larvae fed on the same plant (cabbage to cabbage, cotton to cotton) or switched to a different plant diet (cabbage to cotton, cotton to cabbage). The experimental design of the performed study is provided in Figure 1.

### 3.1. Analysis of 16S rRNA

**Figure 2:**
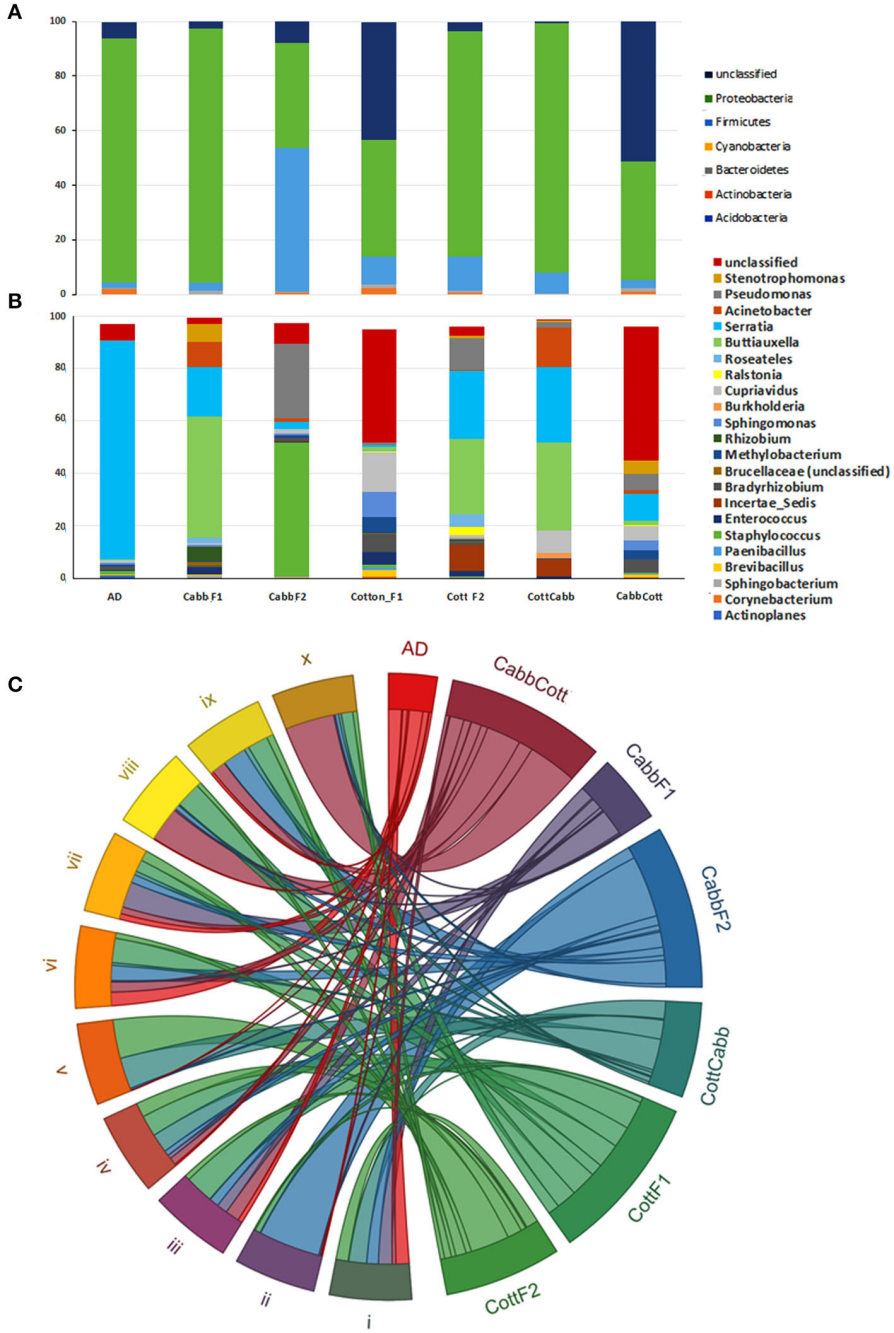
*S. littoralis* larvae gut microbiota across the samples. Distribution and transition of most abundant phylum **(A)** and taxa level **(B)** microbiota during feeding; Top 10 most abundant bacterial classes shared after different feeding **(C)** [i) *Gammaproteobacteria*, ii) *Bacilli*, iii) *Alphaproteobacteria*, iv) *Betaproteobacteria*, v) *Erysipelotrichia*, vi) *Actinobacteria*, vii) *Sphingobacteriia*, viii) *Clostridia*, ix) *Flavobacteriia*, and x) *Bacteroidia*].

High-throughput 16S rRNA sequencing data were produced for the *S. littoralis* larval gut microbial community composition analysis before insect rearing on plant material (Artificial diet; F0) and after rearing on plant material at F1 (CottF1 and CabbF1) and F2 (CottF2, CottCabb, CabbF2, and CabbCott) (Figure 1). A total of 2,623,491 reads were generated across the 21 samples (Supplementary Excel 1). An average of 125,532 reads per sample was found after removing sequences containing ambiguous bases (Ns), chimeric sequences, low Phred quality score (<30), long homopolymer stretches, and some host contaminants. Clustering at 97% sequence identity has generated 3,184 unique clusters, including 2,607,902 reads. Sequence reads were clustered *de novo* into groups based on their sequence similarity to characterize the sequenced microbiome. Subsequently, the centroids of these similarity groups were classified as operational taxonomic units (OTUs) using neighbor-joining algorithms. In 16S analysis, 18 phyla, 44 classes, 84 orders, 153 families, and 255 genera were identified from different samples (Figures 2A, B, Supplementary Excel 2). *Proteobacteria, Firmicutes, Cyanobacteria, Bacteroidetes, Actinobacteria*, and *Acidobacteria* are the most abundant phyla (Figure 2A), and *Gammaproteobacteria, Bacilli, Alphaproteobacteria, Betaproteobacteria, Erysipelotrichia, Actinobacteria, Sphingobacteriia, Clostridia, Flavobacteriia*, and *Bacteroidia* are the top 10 most abundant bacterial classes across the larval gut samples (Figure 2C). The relative abundance of OTUs with an abundance cutoff of 5000 reads across all the feeding treatments was represented as a heatmap (Supplementary Figure 1).

### 3.2. *S. littoralis* larval gut microbial diversity and community shift with and without diet transition

#### 3.2.1. Beta diversity

**Figure 3:**
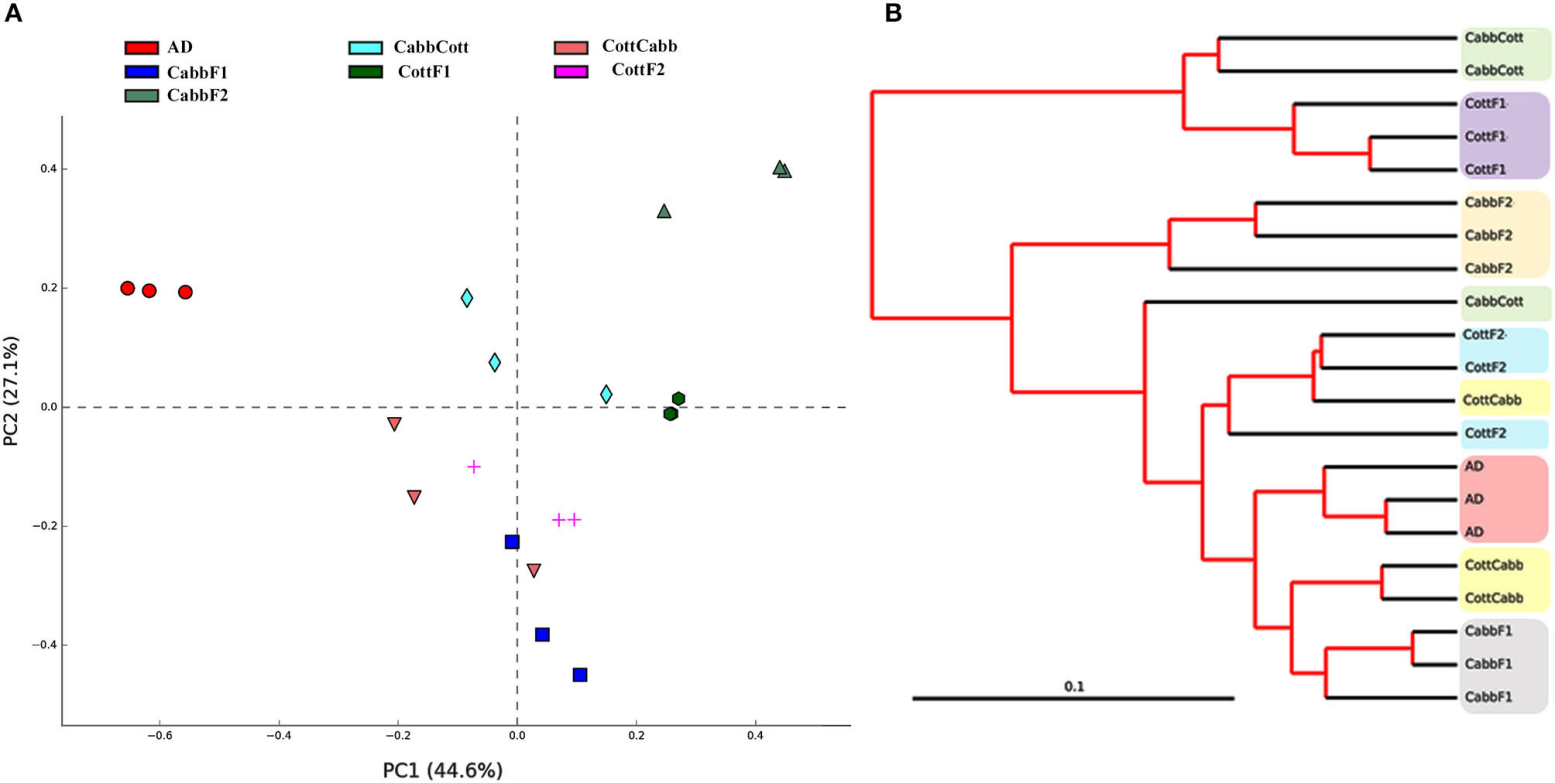
Beta diversity analysis. **(A)** Principal coordinate analysis (PCoA); **(B)** clustering analysis of *S. littoralis* gut microbiome samples to explore the compositional differences of the microbiome.

Principal coordinate analysis (PCoA) and clustering analysis were performed to identify significant compositional variability of the microbiome samples (beta diversity). PCoA plot analysis suggested the presence of three significant clusters of samples based on microbiome composition: artificial diet cluster, CabbF1-CottCabb cluster, and CabbF2-CottF1-CabbCott-CottF2 cluster. The microbiome composition of each metagenome was represented in colored dots (Figure 3A), and the distance among samples represented variations in the bacterial communities. PCoA plot showed that the bacterial assemblage after diet transition (CottCabb and CabbCott) was more similar to the corresponding plant diet at F1 generation, such as CottCabb samples were gathered in the same cluster with CabbF1 samples. Similarly, CabbCott samples were positioned in proximity to CottF1. However, larvae fed on an artificial diet showed distinct microbial assemblage in their gut from those fed on plants.

Furthermore, clustering analysis was performed to ensure the position of samples in clusters complemented by PCoA analysis. Clustering showed artificial diet, CabbF1, and CabbF2, in separate clusters, similar to PCoA analysis (Figure 3B). Two samples of CottCabb were laid next to the CabbF1 cluster, and two samples of CabbCott were laid next to the CottF1 cluster, whereas the third samples of CottCabb and CabbCott were laid close to CottF2. However, the third sample, CabbCott, was placed closer to the cluster made up of CottF2, CottCabb, artificial diet, and CabbF1. Even gut bacterial composition differences were detected within two successive generations after feeding the same plant. PERMANOVA analysis (variable group; Bray–Curtis- Pseudo-f statistic = 3.16, *p*-value = 0.00001; D_0.5 UniFrac = Pseudo-f statistic = 3.03, *p*-value = 0.00002) also confirmed significant differences among some of these samples, such as artificial diet vs. most of the plant samples, CabbF1 vs. CabbF2, CottF1 vs. CottF2, etc. (Supplementary Excel 3). Hence, *S. littoralis* larval gut bacteriome was strongly influenced by ingested food and produced a quick shift in diversity and abundance after the diet switch.

#### 3.2.2. Alpha diversity

**Figure 4:**
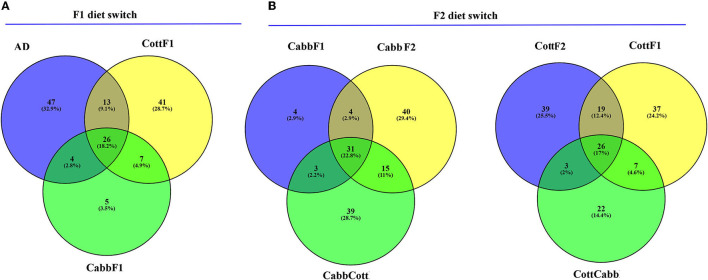
Venn diagrams depict the shared and unique OTUs (non-chimeric and picked at a 97% identity level) within *S. littoralis* larvae after the F1 and F2 transgenerational diet switch. **(A)** Comparison between OTUs (≥2) among cotton, cabbage, and artificial diet-fed larvae. **(B)** Comparison between OTUs (≥2) among cotton (F1), cotton-cabbage (F2), cotton (F2) and cabbage (F1), cabbage-cotton (F2), and cabbage (F2) fed *S. littoralis* larvae. Please refer to the Supplementary Excels 4–6 for further details.

Alpha diversity is essential to understanding specific diet associations with bacterial species. Therefore, Chao1, phylogenetic diversity (PD), and Simpson and Shannon indices were calculated, and the statistical significance was determined using Kruskal–Wallis one-way ANOVA by ranks test as indicated by different letters (Table 1). A higher Chao1 index shows higher species richness in the microbiome, whereas high PD values of samples show low species relatedness. PD measures evolutionary history based on biodiversity rather than species counts. In our study, CabbF1 (195.88, 2.84), CottF2 (192.88, 3.01), and CottF1 (115.82, 3.47) samples showed the highest and lowest species richness of samples and corresponding relatedness, whereas artificial diet, CabbCott, CabbF2, and CottCabb samples showed moderate richness and relatedness of species. Two different assumptions based on the Shannon (randomness) and Simpson (abundance) indices were estimated for species diversity of different diet samples. High Shannon and low Simpson index values of samples such as CabbCott (2.99, 0.73), CabbF1 (3.10, 0.75), CottF1 (3.12, 0.75), and CottF2 (3.54, 0.85) reflect the higher microbiome diversity (Table 1). Captivating dynamics in the succession of bacteriome acquisition were observed after F1 and F2 transgenerational diet switches (Figure 4). The unique OTUs in artificial diet (47) were more than in cotton (CottF1- 41) and cabbage (CabbF1-5) in the F1 generation. In contrast, the number of unique OTUs increased in the F2 generation larvae fed on the same cabbage diet cabbage (CabbF2-40). However, the diet switch at F2 reduced unique OTUs in cotton to cabbage (CottCabb-22) fed larval gut samples (Figure 4). A total of 26 OTUs were shared between artificial diet, cotton-, and cabbage-fed larval samples in the F1 diet switch. After the F2 diet switch, the number of shared OTUs between samples did not vary much (31,26) (Figure 4). Between artificial diet, CottF1, and CabbF1 (F1 diet switch, Figure 4, Supplementary Excel 4), members of Proteobacteria (i.e., *Serratia, Bradyrhizobium, Sphingomonas, Methylobacterium, Pseudomonas, Stenotrophomonas*, and *Paracoccus*), *Bacteroidetes* (i.e., *Flavobacterium* and *Chitinophaga*), *Actinobacteria* (i.e., *Propionibacterium* and *Corynebacterium*), and *Firmicutes* (i.e., *Brevibacillus, Enterococcus*, and *Paenibacillus*) were shared. Similarly, for the F2 diet switch from CabbF1 to CabbF2 and CabbCott, the shared members were Proteobacteria (i.e., *Buttiauxella, Serratia, Acinetobacter, Stenotrophomonas, Rhizobium, Sphingomonas*, and *Pseudomonas*), *Bacteroidetes* (i.e., *Prevotella and Flavobacterium*), *Actinobacteria* (i.e., *Corynebacterium, Propionibacterium*, and *Brevibacterium*), and *Firmicutes* (i.e., *Bacillus* and *Brevibacillus*) (Supplementary Excel 5). The other diet switch between CottF1 to CottF2 and CottCabb in the F2 generation also reveals similar sharing of the bacteriome between samples, such as Proteobacteria (i.e., *Sphingomonas, Bradyrhizobium, Methylobacterium*, and *Serratia*), Bacteroidetes (i.e., *Sphingobacterium*), *Actinobacteria* (i.e., *Micrococcus* and *Corynebacterium*), and *Firmicutes* (i.e., *Bacillus*) (Supplementary Excel 6).

**Table 1:** Estimated alpha-diversity indices for different diet samples for bacterial abundance and consistency.

**Feeding experiment**	**Species richness indices**		**Species diversity indices**		**Number of OTUs**	**Dominated OTUs (Description order as per read count: high to low)**
	**Chao1** ^$^	**PD tree** ^$^	**Shannon** ^#^	**Simpson** ^*^	**Avg. read count (**>**0)**	
AD	152,06	2.97	1.45^a^	0.33^a^	138	*Serratia, Bradyrhizobium, and Staphylococcus*
CabbCott	153,19	3.45	2.99^b, c, d^	0.73^b^	93	*Serratia, Pseudomonas, and Cupriavidus*
CabbF1	195,88	2.84	3.10^b, c, d^	0.75 ^b, c^	85	*Buttiauxella, Serratia, and Acinetobacter*
CabbF2	162,65	3.14	2.64^b^	0.72^b^	97	*Staphylococcus, Serratia, and Pseudomonas*
CottCabb	168,61	2.61	2.57^b^	0.73^b^	65	*Buttiauxella, Serratia, and Acinetobacter*
CottF1	115,82	3.47	3.12^c^	0.75^b^	84	*Cupriavidus, Sphingomonas, and Bradyrhizobium*
CottF2	192,88	3.01	3.54^d^	0.85^c^	98	*Buttiauxella, Serratia, and Pseudomonas*

Different letters indicate a significant difference (*p*-value < 0.05). As per the Kruskal–Wallis test, the group differences are insignificant for Chao1 and PD tree. ^#^Kruskal–Wallis *p*-value = 0.02; ^*^Kruskal–Wallis *p*-value = 0.05; ^$^Kruskal–Wallis *p*-value = NS.

### 3.3. Differential abundance analysis of *S. littoralis* larval gut microbiome

**Figure 5:**
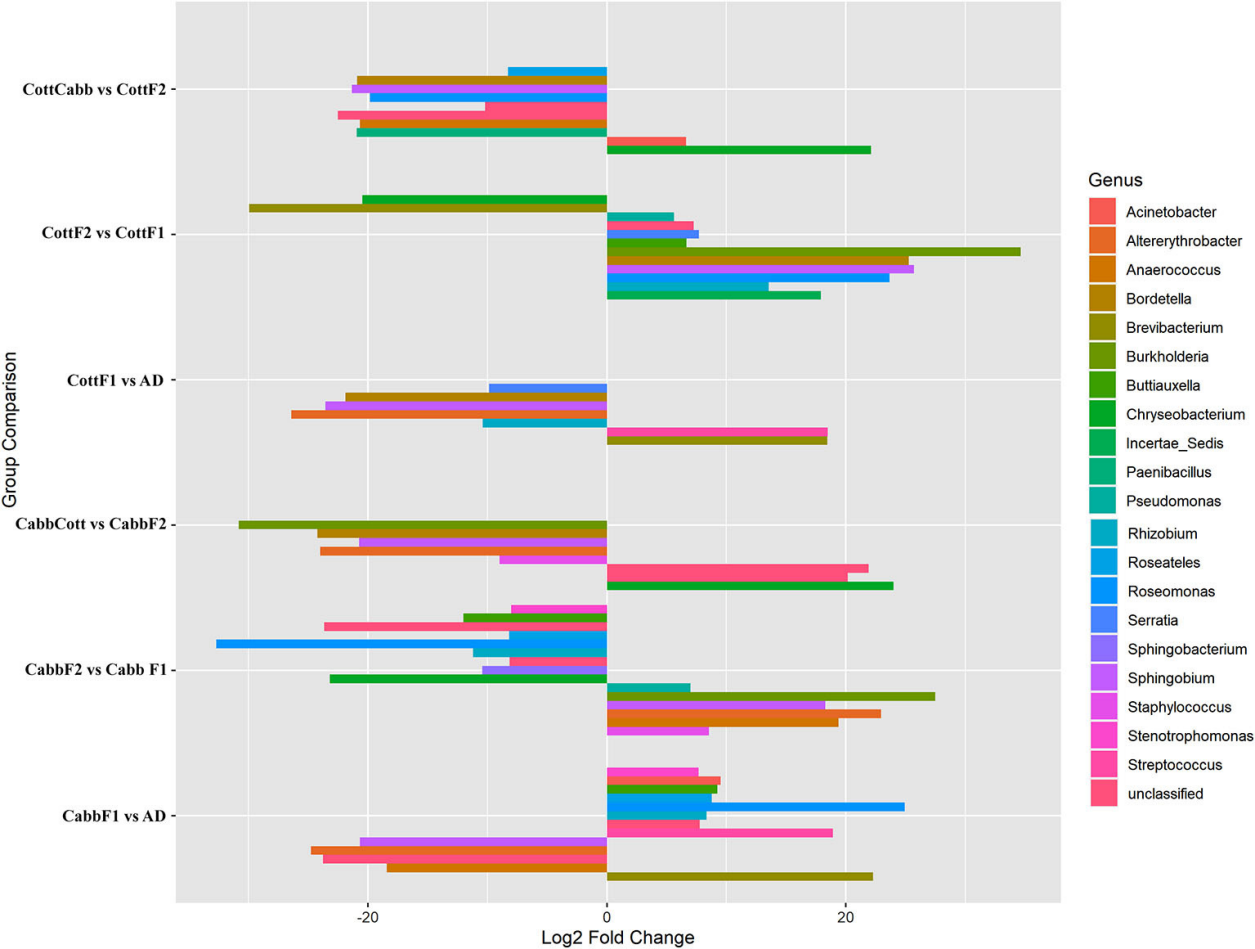
Pairwise differential abundance analysis of gut bacteriome (OTUs) of *S. littoralis* after diet switch experiments. Differentially abundant OTUs (*p* < 0.05) and log2FoldChange are shown concerning feeding experiments. The X-axis represents “log2 Fold Change” values indicating the increase and decrease of genus abundance. The Y-axis represents the assigned OTUs to the genus for the feed experiment group.

DAA based on normalized OTU counts was performed using the R package (DEseq2) to explore significant associations between taxa abundance and feeding experiments. DAA revealed a considerable microbial community shift concerning diet switch. In this study, genus level was focused on reporting and discussing bacterial communities across the diet switch experiments (artificial diet-CabbF1, CabbF1-CabbF2, CabbF2-CabbCott; artificial diet-CottF1, CottF1-CottF2, and CottF2-CottCabb) at significance level (*p*_adjust_ < 0.05) (Figure 5; Supplementary Excel 7).

#### 3.3.1. Cabbage diet effect

During the diet switch from artificial diet to Cabb (F1), the relative abundances of bacterial genera *Brevibacterium* (p_Actinobacteria), *Streptococcus* (p_Firmicutes), and *Rhizobium, Roseomonas, Roseateles, Buttiauxella, Acinetobacter*, and *Stenotrophomonas (*p_Proteobacteria*)* were increased significantly, whereas the abundances of genera *Anaerococcus* (p_Firmicutes)*, Sphingobium*, and *Altererythrobacter* (p_Proteobacteria) were decreased at an approximately similar magnitude. However, *Roseomonas* (log_2_FC = 24.95), *Brevibacterium* (log_2_FC = 22.28), and *Streptococcus* (log_2_FC = 18.94) had a relatively higher abundance than other genera. Interestingly, the abundance of *Rhizobium, Roseomonas, Roseateles, Buttiauxella*, and *Stenotrophomonas* genera was among the differentially abundant genera between artificial diet and CabbF1 indicating their requirement to maintain the internal homeostasis of the larval gut microbiome during diet switch. Another interesting microbial community shift was observed for the CabbF1 to CabbF2 feeding experiment. For instance, bacterial genera including *Sphingobacterium* and *Chryseobacterium* (p_Bacteroidetes), *Rhizobium, Roseomonas, Roseateles, Buttiauxella*, and *Stenotrophomonas* (p-proteobacteria) abundance drastically decreased for diet change CabbF1 to CabbF2. In the CabbF2, the differential abundances of *Staphylococcus* and *Anaerococcus* (p-Firmicutes), *Altererythrobacter, Sphingobium, Burkholderia*, and *Pseudomonas* genera (p-Proteobacteria) were increased. *Altererythrobacter* (log_2_FC = 22.95) *and Burkholderia* (log_2_FC = 27.48) had the highest log_2_FoldChange value among all differentially increased genera (Figure 5; Supplementary Excel 7).

When CabbF1-fed *S. littoralis* larvae were reared on a cotton plant (CabbF1 to CabbCott), the abundances of *Staphylococcus* (log_2_FC = −8.98) (p-Firmicutes), *Altererythrobacter* (log_2_FC = −23.98), *Sphingobium* (log_2_FC = −20.73), *Bordetella* (log_2_FC = −24.24), and *Burkholderia* (log_2_FC = −30.83) (p-Proteobacteria) were reduced. Instead, bacterial families belonging to *Flavobacteriaceae, Caulobacteraceae*, and *Comamonadaceae* (p-Proteobacteria) became more abundant during the sudden diet change from cabbage to the cotton plant (Figure 5; Supplementary Excel 7). Nevertheless, these bacterial consortia may be required to digest the cotton leaves better.

#### 3.3.2. Cotton diet effect

Like the cabbage diet, diet alteration experiments were also performed on the cotton. The shift of bacterial communities from an artificial diet to CottF1 was similar to the cabbage diet transition (artificial diet to CabbF1). The abundance of two genera *Brevibacterium* (log_2_FC: 18.44) (p-Actinobacteria) and *Streptococcus* (log_2_FC: 18.47) (p-Firmicutes) increased significantly, whereas the abundances of genera *Altererythrobacter* (log_2_FC: −26.41) and *Sphingobium* (log_2_FC: −23.57) (p-Proteobacteria) were decreased after diet transition from artificial diet to cabbage. The abundance of *Rhizobium* (log_2_FC: −10.39)*, Bordetella* (log_2_FC: −21.88), and *Serratia* (log_2_FC: −9.85) also decreased during this diet transition. Interestingly, the abundance of *Rhizobium* was decreased for the diet transition from artificial diet to cotton, whereas *Rhizobium* abundance (log_2_FC: −10.39) was increased during the artificial diet to cabbage diet transition.

In the diet transition from CottF1 to CottF2 experiment, the abundances of *Brevibacterium* (log_2_FC: −29.93) (p-Actinobacteria) and *Chryseobacterium* (log2FC: −20.48) (p-Bacteroidetes) were decreased significantly whereas the abundances *Rhizobium* (log_2_FC: 13.54), *Roseomonas* (log_2_FC: 23.67), *Sphingobium* (log_2_FC: 25.71), *Bordetella* (log_2_FC: 25.29), *Burkholderia* (log_2_FC: 4.63), *Buttiauxella* (log_2_FC: 6.67), *Serratia* (log_2_FC: 7.70), *Pseudomonas* (log_2_FC: 5.63) (p-Proteobacteria), and *Incertae_Sedis* (log_2_FC: 17.93) (p-Firmicutes) were increased in comparison with first feeding generation of cotton (CottF1) (Table 2). In the different diet transition (CottF1-CottCabb) experiments, the abundances of *Chryseobacterium* (log_2_FC: 22.1) (p-Bacteroidetes) and *Acinetobacter* (log_2_FC: 6.61) (p-Proteobacteria) were increased significantly whereas the abundances of *Roseomonas* (log_2_FC: −19.83), *Sphingobium* (log_2_FC: −21.34), *Bordetella* (log_2_FC: −20.91), *Roseateles* (log_2_FC: −8.27) (p-Proteobacteria), and *Paenibacillus* (log_2_FC: −20.94) and *Anaerococcus* (log_2_FC: −20.67) (p-Firmicutes) were decreased when cotton feeding generation (CottF1) was forced to cabbage feeding (CottCabb).

**Table 2:** Log2 fold change of differentially abundant OTUs (p_adjust_ < 0.05) among feeding experiments [artificial diet (AD) to plant diet transition (Cabb/Cott), same plant diet transition (Cabb-Cabb/Cott-Cott), and different diet transition (Cabb-Cott/Cott-Cabb)].

**OUT (Phylum/Genus)**	**Log**_**2**_ **fold changes of OTUs in pairwise comparison**					
	**AD-CabbF1**	**AD-CottF1**	**CabbF1-CabbF2**	**CottF1-CottF2**	**CabbF2-CabbCott**	**CottF2-CottCabb**
p_Actinobacteria_g_*Brevibacterium*	++	++	N.D.	—	ND	ND
p_Firmicutes_g_*Streptococcus*	++	++	ND	ND	ND	ND
p_Proteobacteria_f_*Brucellaceae*_g_unclassified	+	ND	ND	-	N.D.	–
p_Proteobacteria_*Rhizobium*	+^*^	–^*^	–^*^	++^*^	ND	ND
p_Proteobacteria_*Roseomonas*	+++	ND	—^*^	+++^*^	N.D.	–
p_Proteobacteria_*Roseateles*	+	N.D.	-	ND	ND	-
p_Proteobacteria_*Buttiauxella*	+	N.D.	- -^*^	+^*^	ND	ND
p_Proteobacteria_g_*Acinetobacter*	+	ND	ND	ND	ND	+
p_Proteobacteria_g_Stenotrophomonas	+	N.D.	-	ND	ND	ND
p_Firmicutes_g_*Anaerococcus*	–	N.D.	++	ND	ND	—
p_Proteobacteria_f_*Caulobacteraceae*_g_unclassified	—	ND	ND	ND	+++^*^	—^*^
p_Proteobacteria_g_*Altererythrobacter*	—	—	+++	N.D.	—	N.D.
p_Proteobacteria_g_*Sphingobium*	—	—	++	+++	—	—
p_Firmicutes_g_*Staphylococcus*	ND	ND	+	N.D.	-	N.D.
p_Proteobacteria_g_*Burkholderia*	ND	ND	+++	+++	—	N.D.
p_Proteobacteria_g_*Pseudomonas*	ND	ND	+	+	ND	ND
p_Bacteroidetes_g_*Chryseobacterium*	ND	ND	—	—	+++	+++
p_Bacteroidetes_g_*Sphingobacterium*	ND	ND	–	ND	ND	ND
p_Proteobacteria_f_*Comamonadaceae*_g_unclassified	ND	ND	—	ND	+++	ND
p_Proteobacteria_g_*Bordetella*	ND	—	N.D.	+++	—	—
p_Proteobacteria_g_*Serratia*	ND	-	N.D.	+	ND	ND
p_Firmicutes_g_*Incertae*_Sedis	ND	ND	ND	++	ND	ND
p_Proteobacteria_f_*Enterobacteriaceae*_g_unclassified	ND	ND	ND	+	ND	ND
p_Firmicutes_g_*Paenibacillus*	ND	ND	ND	ND	ND	—

Bold values show similar trend, whereas asterisk ^*^shows opposite trend within experimental group; Log2FC: log2 fold change; “+/-,” Low log2FC(= >5 and < =10); “++/–,” medium log2FC(= >10 and < =20); “+++/—,” high log2FC(>20); ND: log2 fold change not detected. For more details, refer to Supplementary Excel 7.

### 3.4. Core gut microbiome

**Figure 6:**
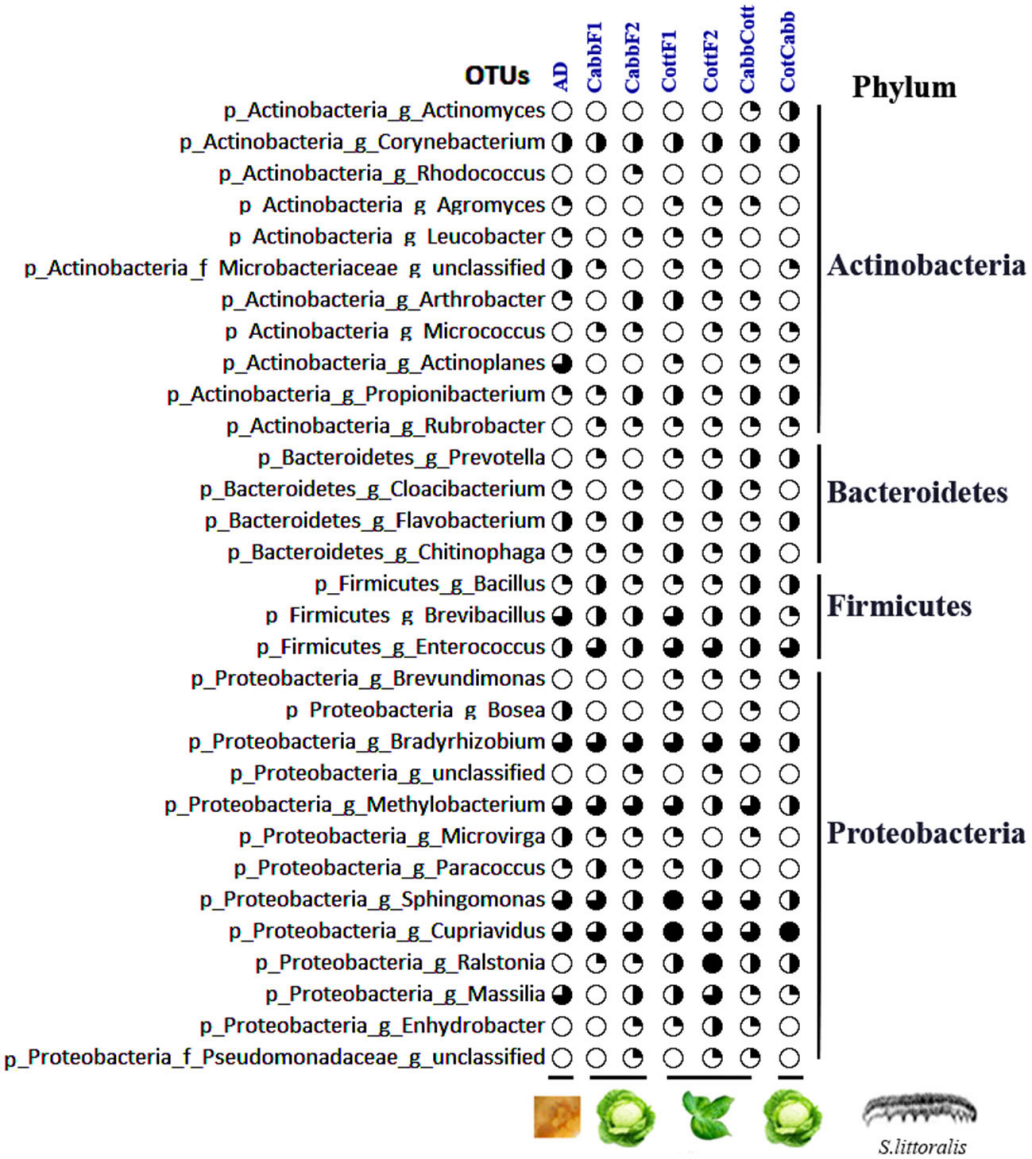
Core gut bacteriome of *S. littoralis* larval gut samples and their relative abundance based on log_10_(normalized read count+1) values (filled part of the circle with black color). DESeq2 normalized read counts are provided in Supplementary Table 1.

The core microbiome in the insect gut performs a conserved function and remains unchanged in varied experimental conditions (Shade and Handelsman, 2012). This study identified non-differentially abundant core OTUs across the sample through DESeq2 normalized read counts of OTUs. The OTUs were considered core communities if those OTUs were present in at least 70% of total samples and listed all phyla along with the normalized read count for each feeding experiment (Figure 6, Supplementary Table 1). Proteobacteria, Actinobacteria, Bacteroidetes, and Firmicutes are the most prominent phyla in the core bacteriome across the samples.

### 3.5. Impact on resident gut microbial metabolic pathways after transgenerational diet switch

**Figure 7:**
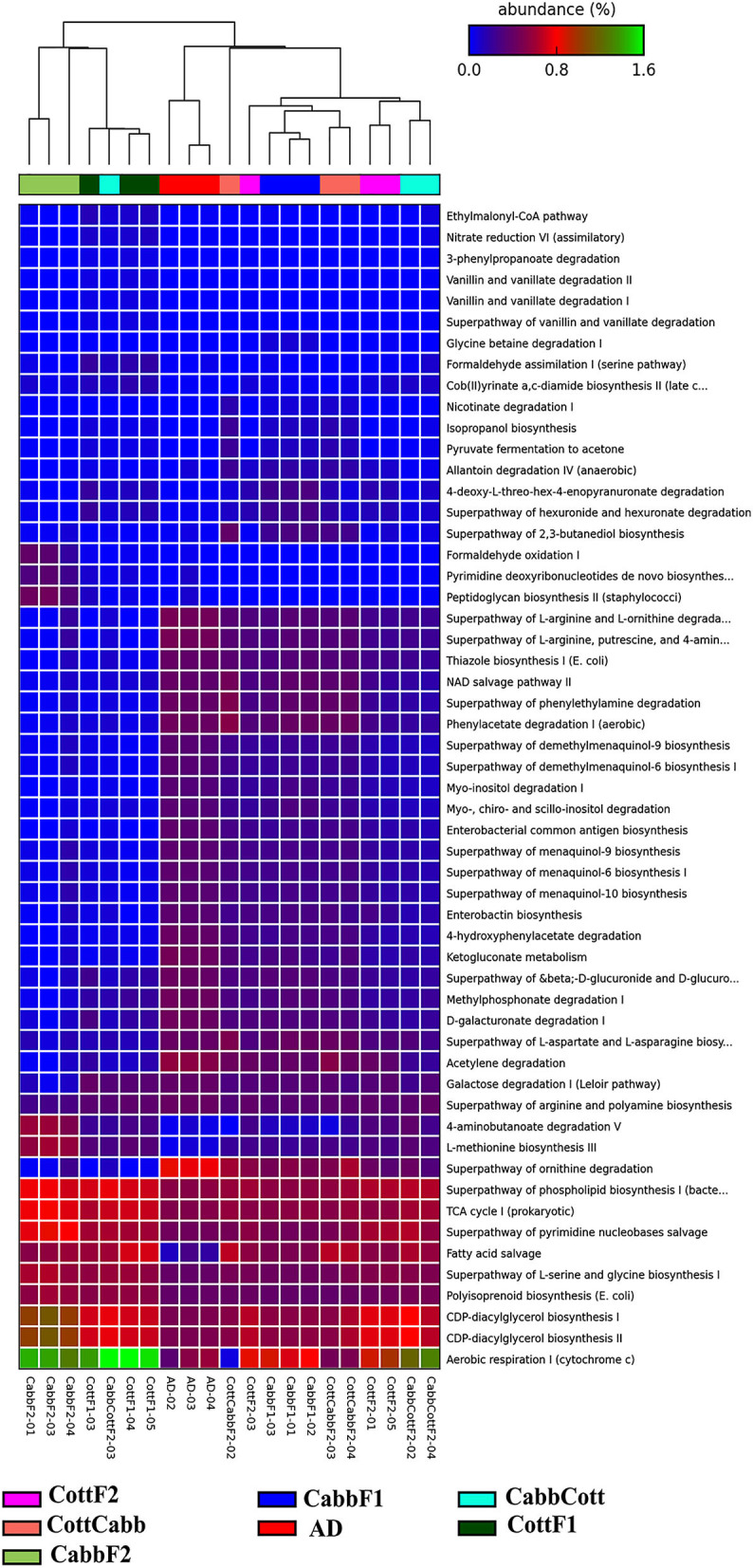
PICRUSt2 predicted significant (*P*-value < 0.001 cutoffs) metabolic pathways across different *S. littoralis* gut samples.

Insect gut bacteriome communities actively participate in metabolic activities (Itoh et al., 2018; Chen et al., 2020, 2021; Ibarra-Juarez et al., 2020; Liu et al., 2022). Therefore, elucidating activated metabolic pathways, especially those associated with the metabolism of dietary components, is crucial to evaluating the relevance underlying the readjustment of bacteriome after a diet switch. PICRUSt2 predicted the metabolic profile of each metagenome to understand the bacterial metabolic shift based on diet (Figure 7; Supplementary Excel 8) and correlated with the differential bacterial taxonomic profile that might be responsible for varied metabolic activities (Supplementary Figure 2; Supplementary Excel 9).

Multi-group statistical analysis was performed through ANOVA and *post hoc* tests using the Tukey–Kramer method to explore differences between multiple group means of feeding experiment. In the pathway prediction analysis, aerobic respiration I (PWY-3781), CDP-diacylglycerol biosynthesis I (PWY-5667), CDP-diacylglycerol biosynthesis II (PWY0-1319), polyisoprenoid biosynthesis (POLYISOPRENSYN -PWY), superpathway of L-serine and glycine biosynthesis I (SER-GLYSYN-PWY), fatty acid salvage (PWY-7094), superpathway of pyrimidine nucleobase salvage (PWY- 7208), superpathway of ornithine degradation (ORNDEG-PWY), TCA cycle I (TCA), superpathway of phospholipid biosynthesis I (PHOSLIPSYN-PWY), 4-aminobutanoate degradation V (PWY-5022), superpathway of arginine and polyamine biosynthesis (ARG+POLYAMINE-SYN), and galactose degradation I (PWY-6317) were among highly abundant metabolic pathways among bacterial community across the samples (Figure 7).

**Figure 8:**
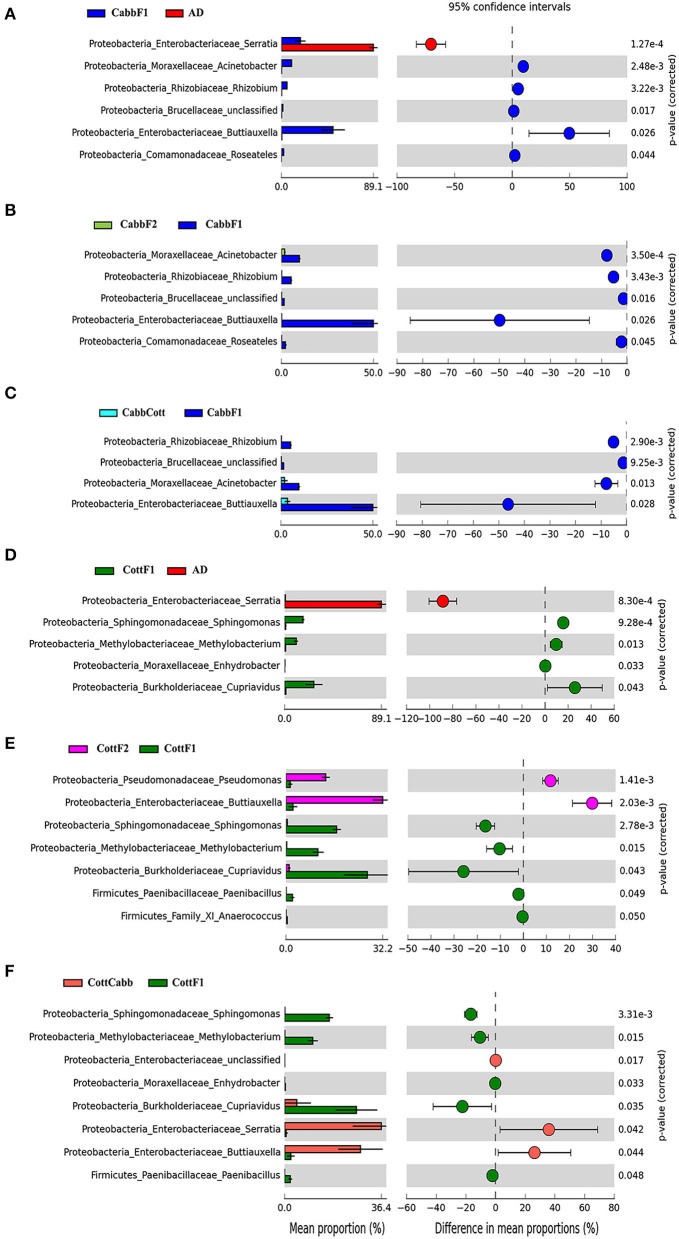
Influence of diet switch on the taxonomic profile of *S. littoralis* larval gut bacteriome revealed after PICRUSt2 analysis. Extended error bar plot showing significant differences between mean proportions of bacterial taxa in artificial diet, cabbage, and cabbage to cotton samples. Corrected *p*-values are shown on the right. Diet change from **(A)** artificial diet to cabbage; **(B)** cabbage to cabbage; **(C)** cabbage to cotton; **(D)** artificial diet to Cotton; **(E)** cotton to cotton; and **(F)** cotton to cabbage. The mean proportion (left side) reflects a possible abundance of microbes possessing each genus, and the dots indicate the difference between mean proportions (effect sizes) for each feature on the right side.

PICRUSt2 functional profile analysis showed several pathways influenced by diet transition, which is correlated with the alteration of the larval gut microbiome (Figure 8; Supplementary Figures 3, 4). A significant difference in taxonomical profile was found in *Spodoptera* gut samples after the diet transition from artificial to cabbage and cotton (Figures 7A, D) that might contribute to the metabolic activities reflected in functional analysis. *Serratia* showed significant-high abundance differences for artificial diet in both comparisons. Interestingly, the functional metabolic profile of larval gut bacteriome shifted even for the same plant diet (CabbF1 to CabbF2, CottF1 to CottF2), feeding in the next generation (Supplementary Figures 5, 6). The top 10 such differentially abundant pathways are listed in Table 3. Bacterial taxonomical profiles for diet shift from cabbage to cabbage, i.e., *Buttiausella, Acinetobacter, Rhizobium*, and *Roseateles*, documented significant mean proportion differences. However, *Pseudomonas, Buttiauxella, Sphingomonas, Methylobacterium, Cupriavidus*, and *Paenibacillus* showed substantial differences in abundance for CottF1 to CottF2 diet shift (Figures 8B, E; Supplementary Figures 5, 6).

**Table 3:** Top 10 significantly different bacterial metabolic pathways in various *Spodoptera* larval gut samples after transgenerational diet switch.

**Comparison**	**Top 10 significantly different metabolic pathways**	***p*-value (corrected)**
AD_CabbF1	Adenosylcobalamin salvage from cobinamide I	3.25E-05
	Adenosylcobalamin salvage from cobinamide II	7.10E-05
	Superpathway of glycolysis and Entner-Doudoroff	8.09E-05
	Adenosylcobalamin biosynthesis from cobyrinate	1.12E-04
	L-lysine biosynthesis III	1.70E-04
	Superpathway of pyrimidine ribonucleosides salvage	2.21E-04
	L-isoleucine biosynthesis IV	3.09E-04
	Glycine betaine degradation I	3.28E-04
	Superpathway of pyrimidine deoxyribonucleoside salvage	3.38E-04
	Superpathway of pyrimidine deoxyribonucleosides degradation	3.43E-04
AD_CottF1	Glucose and glucose-1-phosphate degradation	1.03E-06
	Thiazole biosynthesis I (*E. coli*)	1.43E-06
	dTDP-N-acetylthomosamine biosynthesis	3.63E-06
	Thiazole biosynthesis II (*Bacillus*)	3.79E-06
	L-isoleucine biosynthesis III	6.52E-06
	5-aminoimidazole ribonucleotide biosynthesis I	8.56E-06
	Superpathway of 5-aminoimidazole ribonucleotide biosynthesis	9.04E-06
	5-aminoimidazole ribonucleotide biosynthesis II	9.04E-06
	Mandelate degradation I	1.09E-05
	Mycolate biosynthesis	1.10E-05
CabbF1_CabbF2	Superpathway of hexuronide and hexuronate degradation	6.09E-05
	L-methionine biosynthesis III	8.98E-05
	Superpathway of salicylate degradation	1.38E-04
	Catechol degradation to and beta;-ketoadipate	1.68E-04
	4-methylcatechol degradation (ortho-cleavage)	1.70E-04
	Aromatic compounds degradation *via* and beta;-ketoadipate	2.00E-04
	Catechol degradation III (ortho-cleavage pathway)	2.00E-04
	Myo-, chiro-, and scillo-inositol degradation	3.58E-04
	Allantoin degradation IV (anaerobic)	4.13E-04
	Myo-inositol degradation I	5.28E-04
CotF1_CottF2	dTDP-N-acetylthomosamine biosynthesis	6.46E-06
	Superpathway of UDP-glucose-derived O-antigen building blocks biosynthesis	3.97E-05
	Superpathway of L-aspartate and L-asparagine biosynthesis	5.83E-05
	Superpathway of tetrahydrofolate biosynthesis	8.49E-05
	Superpathway of tetrahydrofolate biosynthesis and salvage	8.67E-05
	L-isoleucine biosynthesis I (from threonine)	1.14E-04
	L-valine biosynthesis	1.14E-04
	Stearate biosynthesis II (bacteria and plants)	1.36E-04
	L-isoleucine biosynthesis II	1.54E-04
	Superpathway of pyridoxal 5′-phosphate biosynthesis	2.12E-04
CabbF2_CabbCott	Superpathway of hexuronide and hexuronate degradation	4.37E-04
	Superpathway of 2,3-butanediol biosynthesis	5.57E-04
	Nicotinate degradation I	7.47E-04
	Allantoin degradation IV (anaerobic)	7.75E-04
	Superpathway of (R,R)-butanediol biosynthesis	1.17E-03
	4-deoxy-L-threo-hex-4-enopyranuronate degradation	1.75E-03
	Glycine betaine degradation I	2.45E-03
	Myo-, chiro- and scillo-inositol degradation	2.58E-03
	Cob(II)yrinate a,c-diamide biosynthesis II	4.59E-03
	Pyruvate fermentation to acetone	5.11E-03
CabbF2_CabbCott	L-methionine biosynthesis III	5.51E-04
	D-galactarate degradation I	2.27E-03
	Superpathway of D-glucarate and D-galactarate degradation	2.27E-03
	CDP-diacylglycerol biosynthesis II	4.25E-03
	CDP-diacylglycerol biosynthesis I	4.25E-03
	Superpathway of polyamine biosynthesis I	4.78E-03
	Methylphosphonate degradation I	5.20E-03
	Superpathway of glucose and xylose degradation	5.35E-03
	Superpathway of arginine and polyamine biosynthesis	6.43E-03
	Peptidoglycan biosynthesis IV (*Enterococcus faecium*)	7.22E-03
CottF1_CottCabb	Mandelate degradation I	6.47E-06
	Superpathway of L-arginine, putrescine, and 4-aminobutanoate degradation	1.08E-05
	Superpathway of L-arginine and L-ornithine degradation	1.08E-05
	Stearate biosynthesis II (bacteria and plants)	1.50E-05
	Enterobacterial common antigen biosynthesis	1.59E-05
	4-hydroxyphenylacetate degradation	1.76E-05
	L-arginine biosynthesis IV (archaebacteria)	2.54E-05
	Superpathway of 5-aminoimidazole ribonucleotide biosynthesis	3.42E-05
	5-aminoimidazole ribonucleotide biosynthesis II	3.42E-05
	Coenzyme A biosynthesis I	4.87E-05
CottF2_CottCabb	Fatty acid salvage	2.32E-04
	Fatty acid and beta;-oxidation I	7.30E-03
	Superpathway of 2,3-butanediol biosynthesis	0.012
	cis-vaccenate biosynthesis	0.016
	Superpathway of phenylethylamine degradation	0.016
	Glycogen degradation I (bacterial)	0.017
	Urate biosynthesis/inosine 5′-phosphate degradation	0.017
	Cob(II)yrinate a,c-diamide biosynthesis II	0.019
	Phenylacetate degradation I (aerobic)	0.019
	TCA cycle IV (2-oxoglutarate decarboxylase)	0.02

CabbF1 to CabbCott and CottF1 to CottCabb fed larval gut bacteriome function (putative) were analyzed to explore metabolic profiles shift associated with different plant diet transitions. After the diet switch, several differential metabolic activities were observed in *S. littoralis* larval gut bacteriome (Supplementary Figures 7, 8). The top 10 differentially abundant pathways for these diet switches are summarized in Table 3. Taxonomic profile for CabbF1 to CabbCott diet shift, genera *Buttiauxella, Acinetobacter*, and *Rhizobium* showed significant abundance differences. In contrast, *Buttiauxella, Paenibacillus, Serratia, Cupriavidus, Methylobacterium*, and *Sphingomonas* showed significant abundance differences for CottF1 to CottCabb diet transition (Figures 8C, F). Furthermore, differentially abundant pathways (top 10) and bacterial taxonomic profile outputs from PICRUSt2 analysis for CottF2 vs. CottCabb and CabbF2 vs. CabbCott comparisons are also represented in Table 3 and Supplementary Figures 9, 10.

## 4. Discussion

Screening generalist insect gut microbial community is essential to comprehend the effect of different diets on resident microbial community structure and function (Mason et al., 2020, 2022; Lv et al., 2021; Hansen and Enders, 2022; Ugwu et al., 2022). However, the impact of transgenerational diet switch on the resident gut microbiome is not yet studied in major generalist pests.

In an earlier study, we found transgenerational effects on larval development based on parental diet, where a matching diet between parental and offspring diet resulted in a better development than a mismatching diet (Rösvik et al., 2020). However, the effect only occurred in one of the tested host plants and not in the other. The transfer of gut microbiota could be one factor influencing this differential transgenerational effect. Therefore, this study was planned to investigate the influence of transgenerational diet changes (artificial diet, cabbage, and cotton) on the gut bacteriome of *S. littoralis* larvae.

The 16S data analysis identified 18 phyla, 44 classes, 84 orders, 153 families, and 255 genera across the tested samples. Proteobacteria (56%), Firmicutes (13%), Actinobacteria (12%), Bacteroidetes (7%), Planctomycetes (2%), and Acidobacteria (1%) are the most abundant among 18 phyla (Figure 2A), which corroborates well with earlier findings on *S. littoralis* bacterial assemblage (Chen et al., 2016). The relative abundance of Proteobacteria was higher in artificial diet, CabbF1, CottF2, and CottCabb than in other samples. The highest number of unclassified phyla and genera was reported for CottF1 and CabbCott samples. However, low abundance and high diversity were observed at the family level across the samples, which is worth further functional investigation using metatranscriptomics, metaproteomics, or culture-based approaches.

Various bacterial species reside inside the *S. littoralis* larval gut that may aid in digesting food and detoxifying phytochemicals (Chen et al., 2016; Paniagua Voirol et al., 2018; Lv et al., 2021). The species richness (Chao1 index) was low in CottF1, which was interesting and needed further functional investigation. The highest PD values were estimated for CottF1, CabbCott, CabbF2, and CottF2 samples suggesting their bacterial community similarity, which was observed by close clustering of these samples after PCoA analysis (Figure 3A). Higher PD values of CottF1 and CabbCott might be associated with diet transition from artificial diet to CottF1 and CabbF1 to CabbCott to obtain higher species richness for cotton diet processing. Moreover, a high percentage of unclassified OTUs were found in CottF1 and CabbCott samples at phylum and genus levels, supporting the sudden appearance of low abundant bacterial species in response to the dietary changes. CottF2, CottF1, CabbF1, and CabbCott had higher Shannon index suggesting higher microbial diversity in those samples. Interestingly, the Simpson index was also increased in these samples indicating the high dominance of specific bacterial genera such as *Serratia, Bradyrhizobium, Staphylococcus, Pseudomonas, Cupriavidus, Buttiauxella, Acinetobacter*, and *Sphingomonas*. Artificial diet and CottF2 samples had the lowest and highest species dominance, respectively. Many of these dominant bacterial species were commonly hosted by lepidopteran insects. For instance, *Pseudomonas* and *Staphylococcus* were reported in >70%, *Serratia* and *Acinetobacter* were documented in >40%, and *Sphingomonas* were present in >20% of lepidopteran species suggesting their conserved role (Paniagua Voirol et al., 2018). Many of these bacteria benefit the insect in digesting the plant material and protecting against pathogens. *Acinetobacter sp*. R7-1 in the Gypsy moth (*Lymantria dispar*) was shown to metabolize phenolic glycosides from aspen (Mason et al., 2016). Similarly, *Pseudomonas* and *Cupriavidus* were documented for their ability to degrade resin acids (Vilanova et al., 2014). *Pseudomonas* was also reported to have antifungal activity against plant pathogenic fungi suggesting their putative protective role in the insect gut (Oh et al., 2015). *Sphingomonas* and *Acinetobacter* were involved in microbiome-mediated insecticide and allelochemical detoxification (Malhotra et al., 2012; Itoh et al., 2018).

### 4.1. Artificial diet to plant feeding: readjusting bacteriome

In many gut microbial studies of insects, an artificial diet is used to generate a reference gut microbiome for comparison and evaluation of experimental conditions such as the influence of plant diet. The simplicity of the artificial diet is advantageous for monitoring the effect of complex plant diets on gut bacterial assemblage. Comparing the transgenerational succession of bacteria in the *S. littoralis* larval gut after the diet switch, it was found that 39% of the OTUs were retained in cotton (CottF1) and 30% in cabbage (CabbF1) fed larvae from artificial diet-fed larvae (F0). Within that, 26% OTUs have been shared among all (artificial diet, CottF1, and CabbF1) larvae (Figure 5A). However, the number of OTUs drastically reduced in *S. littoralis* larval gut after cabbage feeding in the F1 generation, which required further investigation. Nevertheless, maternally transmitted bacteria through egg (or vertically transmitted from mother to offspring) from F0 to F1 generation might perhaps stabilize host–microbe interaction and support co-evolution.

Furthermore, *Brevibacterium* and *Streptococcus* were substantially increased in abundance on cabbage (log_2_FC: 22.28, 18.94) and cotton (log_2_FC: 18.44, 18.47) fed larvae (F1) compared to artificial diet-fed ones (F0). *Brevibacterium frigoritolerans* is an entomopathogen probably acquired during cabbage and cotton feeding (Selvakumar et al., 2011). Similarly, *Streptococcus* was reported for a broad range of biological processes such as food degradation, nutrient absorption, probiotics, and the fight against pathogenic organisms in animals, plants, and insects (Mundt, 1982). Hence, their higher abundance in plant diet and acquisition in *S. littoralis* larval gut is understandable. Furthermore, *Roseomonas* was highly abundant in the cabbage diet. This strain was reported to aid insecticidal resistance (Vijayakumar et al., 2018). It is worth following up if *Roseomonas* aids *Spodoptera* larvae to feed on isothiocyanate-containing crucifers such as cabbage (Wadleigh and Yu, 1988). Interestingly, *Altererythrobacter* and *Sphingobium* (p-Proteobacteria) were reduced significantly after feeding on cabbage (log_2_FC: −24.78, −20.66) and cotton (log_2_FC: −26.41, −23.57) diet (Figure 5). *Altererythrobacter* was reported to have algicidal activities that could control microalgae proliferation and even lyse them (Lei et al., 2014). Hence, they might play a similar protective role against microalgae in the *S. littoralis* larval gut while feeding on an artificial diet, an attractive, nutritious substance for many microorganisms. The role of *Sphingobium* in insect gut is not very clear. However, an increased abundance of *Sphingobium* was reported in Silkworm (*Bombyx mori L*.) against Chloramphenicol (CAM) and vancomycin (VCM) treatment (Guannan, 2020). The genus *Rhizobium* showed an interesting twist after the diet change; the abundance increased in cabbage (Log2FC: 8.35), whereas it reduced in the cotton diet (Log2FC: −10.39). The presence of *Rhizobium* in insect gut may play a crucial role in fulfilling the nutritional nitrogen requirements (Nardi et al., 2002). However, it is hard to rationale the reason for varied *Rhizobium* abundance in cabbage and cotton diet-fed larvae (F1) without further experimental validation.

The putative functional profile of *Spodoptera* larval gut microbiome on different diets revealed modifications in metabolic pathways. For instance, *Serratia* species produced extracellular proteases, chitinases, and other tissue-destructive enzymes that were highly abundant in the artificial diet-fed larval gut (Figure 7A) and might be required to digest the artificial diet, which was not entirely phytochemical-free. *Serratia* was also reported to utilize pectinolytic, xylanolytic, and polysaccharides in the gut of *Bombyx mori* (Prem Anand et al., 2010). Interestingly, the genus *Cupriavidus* showed a higher abundance difference in cotton-fed larvae gut and were known to degrade and recycle allantoin, a nitrogen-rich source derived from purine degradation (Vogels and Van der Drift, 1976). *Cupriavidus* were also documented in the moth, *Retinia resinella*, contributing to the degradation of specific resin acids or diterpenes (Vilanova et al., 2014). GDP-mannose-derived O-antigen building blocks biosynthesis is an abundant metabolic pathway in larval bacteriomes fed on plant diets. It is an essential constituent of lipopolysaccharides to maintain membrane integrity and protection against chemical attacks and animal immune systems (Samuel and Reeves, 2003). Furthermore, protocatechuate degradation pathways were significantly abundant in larval bacteriomes fed on plant diets, probably aiding secondary metabolite degradation (Segers et al., 2017). Protocatechuate is a crucial intermediate metabolite in the microbial degradation of various aromatic compounds, including phthalates, hydroxybenzoates, and lignin-derived aromatic compounds. Later, protocatechuate or catechol is degraded *via* the β-ketoadipate pathway to succinyl-CoA and acetyl-CoA as the sole carbon and energy sources for growth (Harwood and Parales, 1996).

### 4.2. Transgenerational host switch: does it alter larval gut bacteriome?

Transgenerational plant diet shift showed readjustments in the *S. littoralis* larval gut bacterial assemblage. Evaluating the transgenerational succession of bacteria in the *S. littoralis* larval gut after the plant diet switch (F1 to F2), it was found that 34% of the OTUs were retained between CottF1-CottCabb (F2) and 29% between CabbF1-CabbCott, which is very similar to F0-F1 diet switch indicating that approximately 1/3 larval gut bacteriome population could be maternally transmitted and retained in the gut. The other 2/3 bacteriome may be transient or horizontally transferred from the lab environment along with the plant diet. It was needless to mention that the observed ratio may vary in nature. Nevertheless, 8 and 10 bacterial OTUs exhibited differential abundance in pairwise comparisons after cabbage to cotton and cotton to cabbage diet shifts, respectively.

Transgenerational diet switch also influences the putative functional metabolic profile of the resident bacteriome. In CabbF1 to CabbCott diet change, only four metabolic pathways, i.e., the super pathway of hexuronide and hexuronate degradation, nicotinate degradation I, allantoin degradation IV, and butanediol biosynthesis, showed a significant difference (Supplementary Figure 7). The presence of hexuronide, hexuronate, and allantoin degradation pathways may suggest the generation of β-D-glucuronosides as detoxification products in the *S. littoralis* larval gut. *Escherichia coli* has the potential to use β-D-glucuronosides and hexuronates D-glucuronate, D-fructuronate, and D-galacturonate as the sole sources of carbon for growth, whereas *Cupriavidus* species can degrade and recycle allantoin as the source of nitrogen (Scolari et al., 2019; Koga et al., 2022). Interestingly, many other pathways were also reported as differentially abundant for CottF1 to CottCabb (Supplementary Figure 8). Such changes in the bacterial metabolic activities might be related to the different host chemistry encountered by the insect after the transgenerational diet switch.

### 4.3. Transgenerational feeding on the same plant: response of larval gut bacteriome

Transgenerational feeding on the same plants also showed changes in the larval gut bacteriome profile. Assessing the transgenerational movement of bacteria in the *Spodoptera* larval gut after the same plant diet feeding (F1 to F2), it was found that 35% of the OTUs were retained between CabbF1-CabbF2 and 45% between CottF1-CottF2, which is comparable to other diet switches and hence, supporting our previous assumption of 1/3 gut bacteriome retention in different generations irrespective of diet and environment. In differential OTU abundance analysis, 15 and 12 OTUs showed differential abundance between CabbF1-CabbF2 and CottF1-CottF2 comparisons, respectively. For instance, *Burkholderia* (log_2_FC: 27.48, 34.63), *Pseudomonas* (log_2_FC: 7.01, 5.63), and *Sphingobium* (log_2_FC: 18.28, 25.71) abundances were increased, whereas *Chryseobacterium* abundance (log_2_FC: −23.18, −20.48) was decreased in both cabbage and cotton diet, respectively. *Chryseobacterium* sp. was first documented from the midgut of the mosquito *Culex quinquefasciatus*, offering a beneficial role for their host, saving axenic larval mosquito development (Kämpfer et al., 2010; Coon et al., 2014; Chen et al., 2015). *Pseudomonas* and *Burkholderia* are well-known for pathogen suppression, nutritional benefits and resistance against insecticides, defense against pathogenic fungi, and nitrogen metabolism in ants (Flury et al., 2016; Kaltenpoth and Flórez, 2020). Decreased abundance of *Chryseobacterium* and increase in *Pseudomonas* and *Burkholderia* may reflect the adaptive fine-tuning in the transgenerational feeding on the same plants. The functional metabolic profile of the *S. littoralis* larval gut bacteriome was also altered after the transgenerational feeding on the same plant. This might be explained through the shifts in bacterial taxonomical profile. It is worth mentioning here that the functional profile of bacterial communities is extrapolated from what is already known from other systems, and hence, it requires functional validation. Nevertheless, the changes in the *Spodoptera* gut bacteriome after transgenerational feeding on the same diet seem very transient and may rely on the microbiome of the host environment.

## 5. Conclusion

This study has demonstrated a considerable bacterial community shift upon transgenerational diet change and identified *S. littoralis* core and differentially abundant larval gut microbial community. However, our analysis also showed that bacteriome composition after feeding on different diets does not follow a predictable trend but is very dynamic and often transient. Nevertheless, *Actinobacteria, Bacteroidetes, Firmicutes*, and *Proteobacteria* were among the most abundant phyla in the core microbiome. Some bacterial genera, namely *Cupriavidus, Sphingomonas, Bradyrhizobium, Enterococcus, Methylobacterium, Ralstonia, Brevibacillus, Massilia, Actinoplanes*, and *Corynebacterium*, were the most abundant and surprisingly resilient to diet change suggesting their conserved role in the gut of *Spodoptera* larvae. Increased or decreased abundance of bacterial communities and the altered metabolic functions of gut bacteriome may indicate the plastic bacterial community in the gut *S. littoralis* larvae, which can be linked to a broad host plant diet and its plastic responses during host plant choice. We also observed an overlapping and unpredictable influence of diet switch on *S. littoralis* larval gut microbial assemblage. One limitation of the present study is the lack of host–microbiome information. Hence, it did not measure the horizontal transfer of bacteria after each diet switch, as lepidopterans often acquire symbionts from their host plant (Caspi-Fluger et al., 2012; Chrostek et al., 2017; Li et al., 2017). Further analysis of the active microbial community using the metatranscriptomics, metaproteomics, or culture-dependent approach and identifying the host plant-associated bacteriome can explain the role of bacterial symbiosis in *S. littoralis* host adaptation. Such studies may ultimately lead to the formulation of superior IPM approaches against agriculturally important pests such as *Spodoptera* spp.

## Data availability statement

The data presented in the study are deposited in the NCBI bioproject repository, accession number PRJNA930181.

## Author contributions

PA, AR, and BH conceptualized the study. BH and PA performed the lab work. AR, BH, and SK performed the data analysis and figure preparation. AR and SK wrote the first draft. AR and PA prepared the final draft. All authors read and approved the final draft.

## Supplementary material

Supplementary Figure 1 Heatmap exhibiting the relative abundance of OTUs with an abundance cutoff of 5000 across all the feeding treatments.

Supplementary Figure 2 Heatmap representing PICRUSt predicted differential bacterial taxonomic profile that might be responsible for varied metabolic activities in the *Spodoptera littoralis* gut microbiome.

Supplementary Figure 3 Metabolic pathways influenced by diet transition from artificial diet to cabbage (F1).

Supplementary Figure 4 Metabolic pathways influenced by diet transition from artificial diet to cotton (F1).

Supplementary Figure 5 Metabolic pathways influenced by diet transition from cabbage F1 to cabbage F2.

Supplementary Figure 6 Metabolic pathways influenced by diet transition from cotton F1 to cotton F2.

Supplementary Figure 7 Metabolic pathways influenced by diet transition from cabbage F1 to cabbage-cotton F2.

Supplementary Figure 8 Metabolic pathways influenced by diet transition from cotton F1 to cotton-cabbage F2.

Supplementary Figure 9 Metabolic pathways influenced by diet transition from cotton-cotton F2 to cotton-cabbage F2.

Supplementary Figure 10 Metabolic pathways influenced by diet transition from cabbage-cabbage F2 to cabbage-cotton F2.

Supplementary Excel 1 Sequence counts.

Supplementary Excel 2 Taxonomy-binned_OTU-count-table_97_sequence-counts.

Supplementary Excel 3 PARMANOVA analysis results.

Supplementary Excel 4 Venn comparison between cotF1_cabF1_AD1.

Supplementary Excel 5 Venn comparison between cabbageF1_cabbage_F2_cabbcott.

Supplementary Excel 6 Venn comparison between cotF1_cotF2_cottcabb.

Supplementary Excel 7 DEseq2 analysis summary.

Supplementary Excel 8 Pathway analysis output using PICRUSt2.

Supplementary Excel 9 Differential taxonomic abundance output from PICRUSt2.

Supplementary Table 1 Core gut bacteriome of *S. littoralis* larvae. Numbers in columns represent the average DESeq2 normalized read count for each feeding experiment.
