# Supplementary figures and images for: Impact of transgenerational host switch on gut bacterial assemblage in generalist pest, Spodoptera littoralis (Lepidoptera: Noctuidae)
Source: Front Microbiol. 2023 Jul 13;14:1172601. doi: 10.3389/fmicb.2023.1172601 (PMC10374326; doi:10.3389/fmicb.2023.1172601)

# Supplementary Figure 1

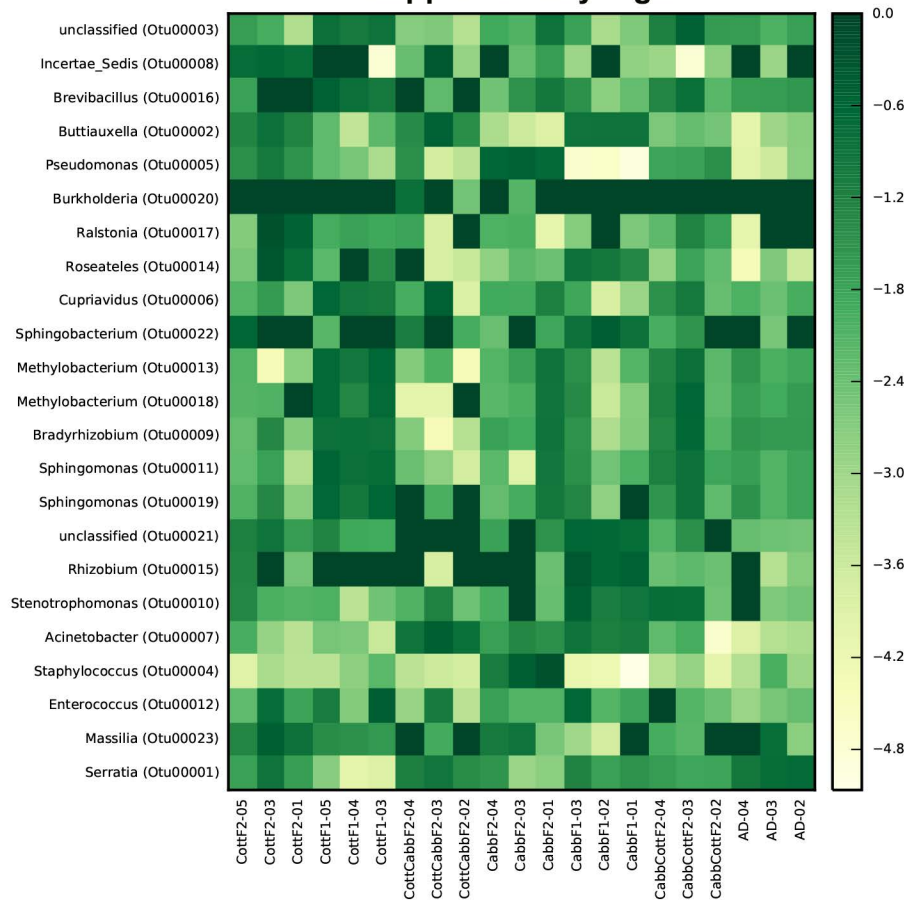

Supplementary Figure 2

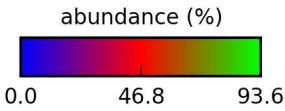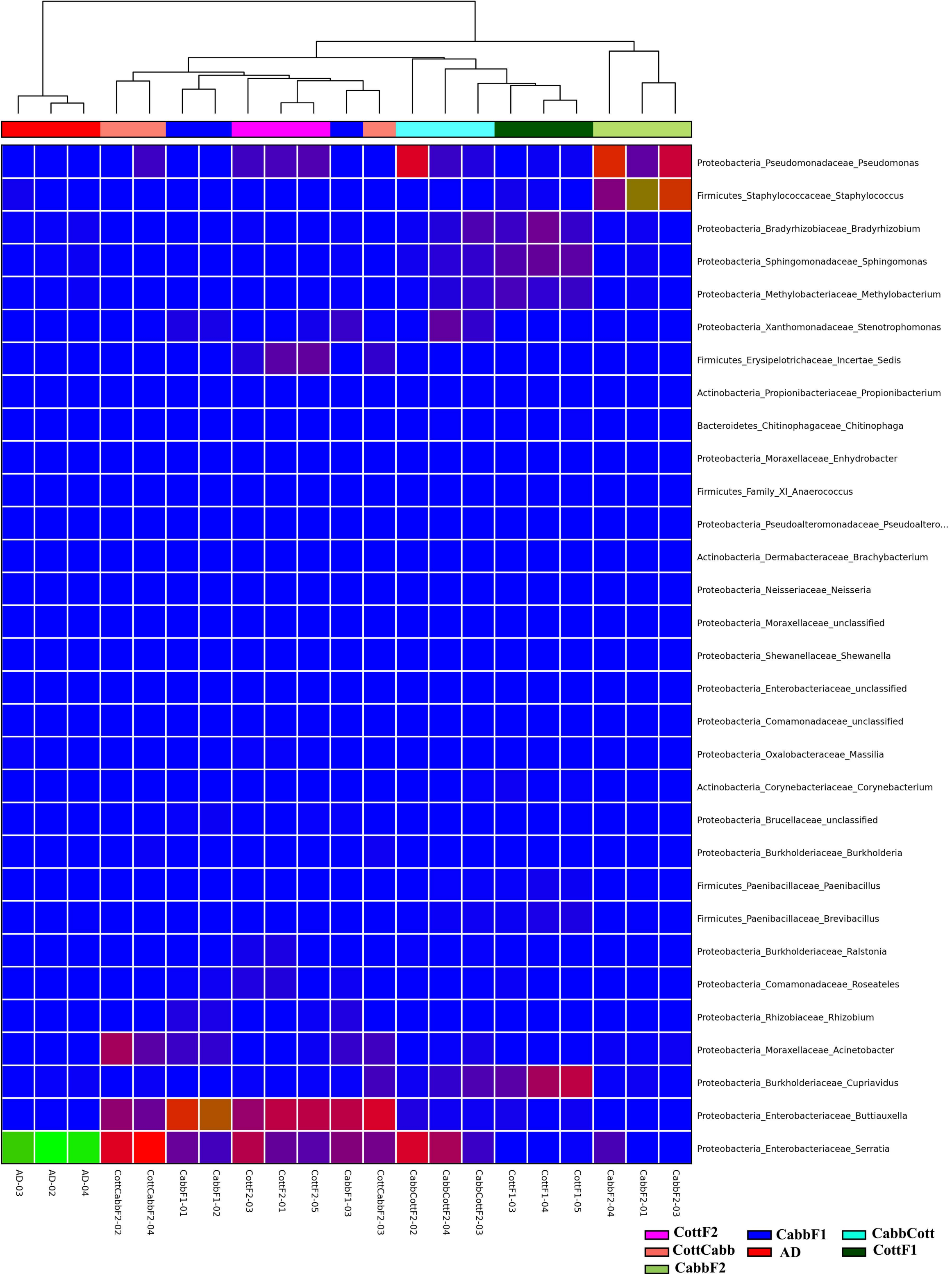

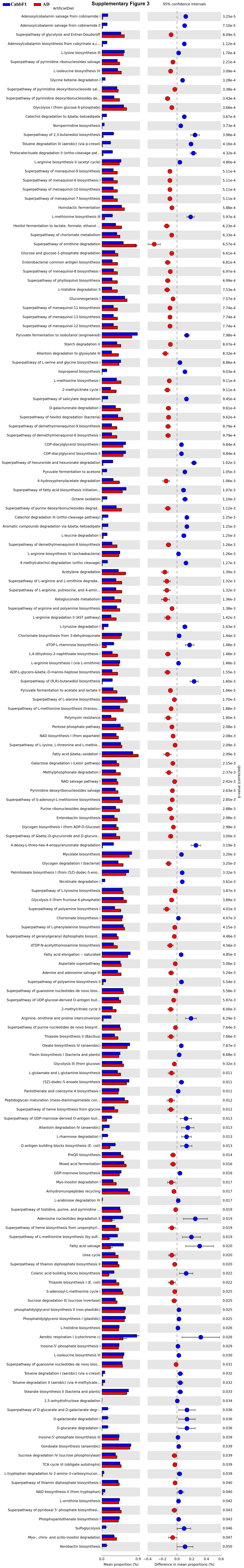

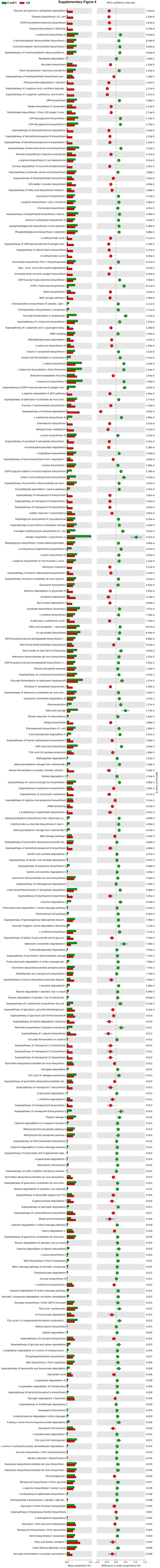

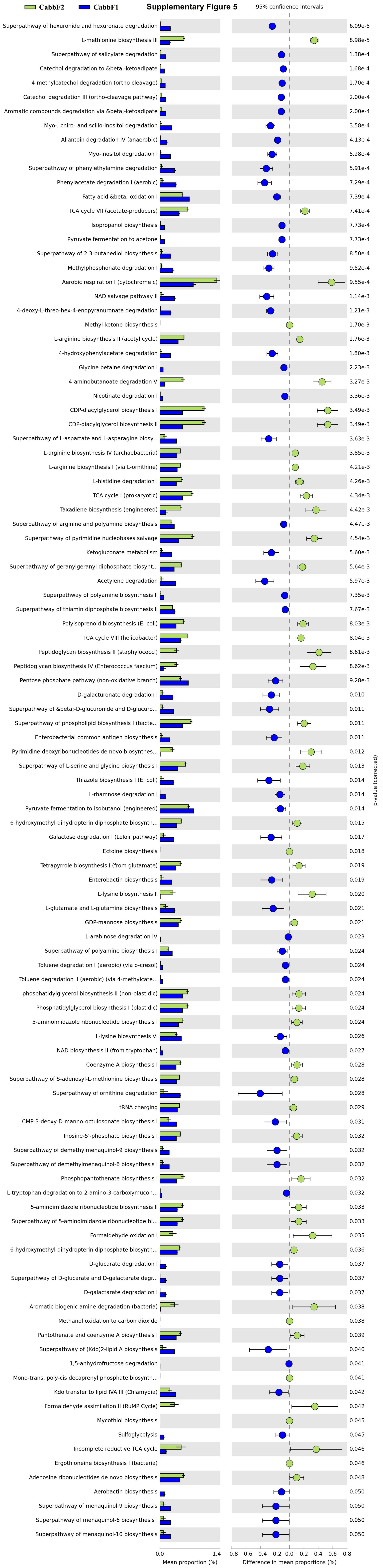

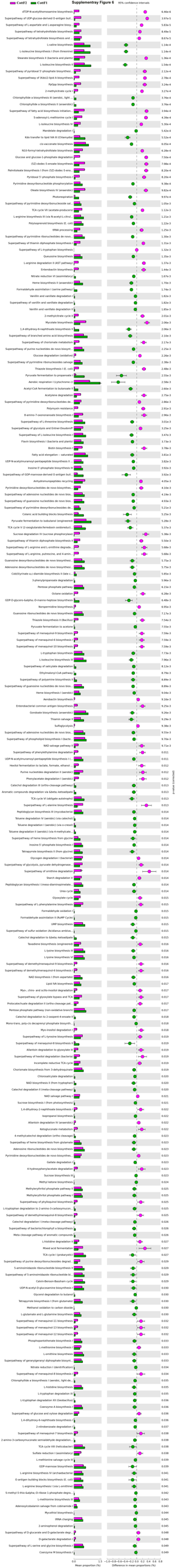

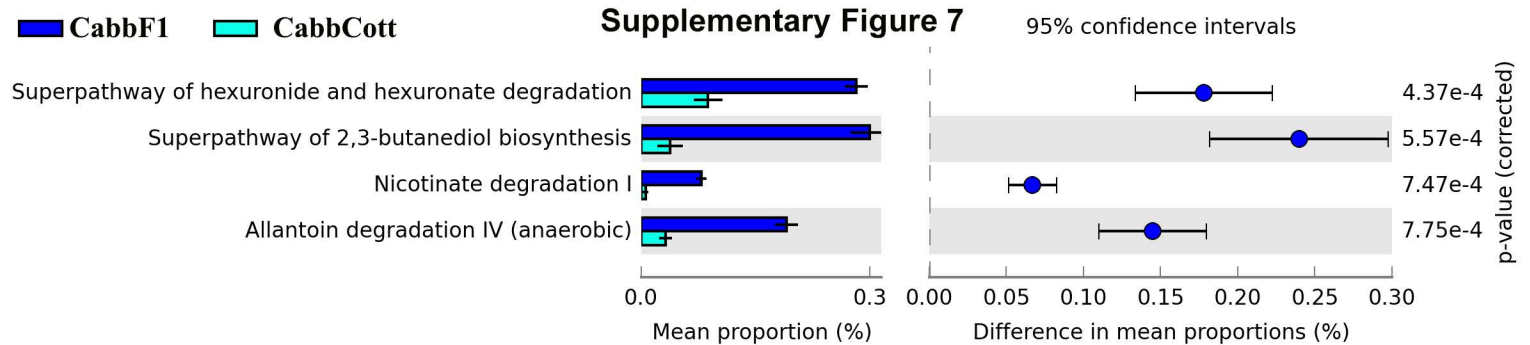

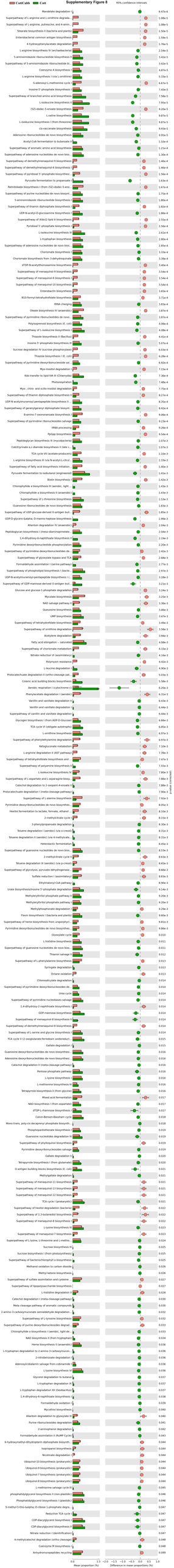

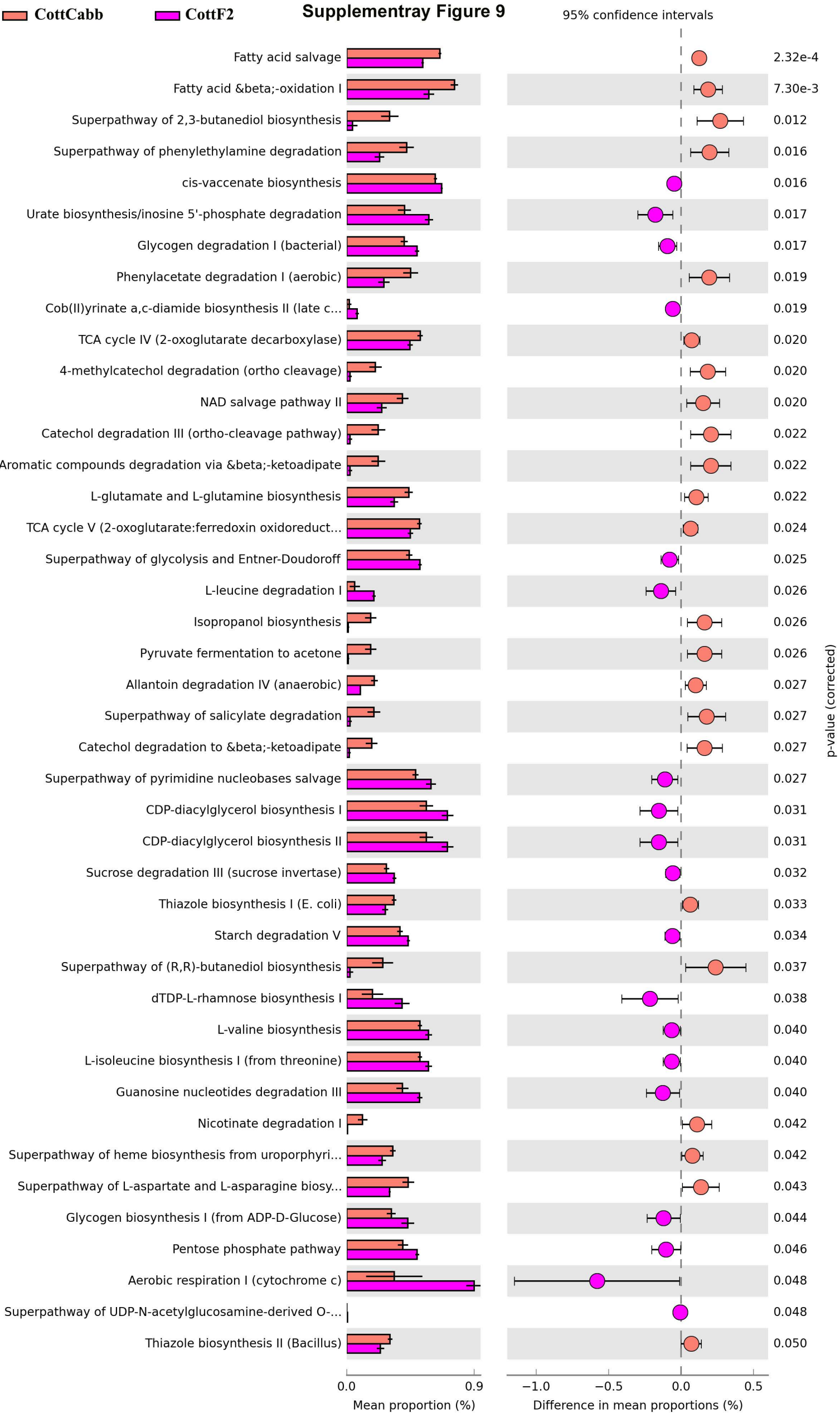

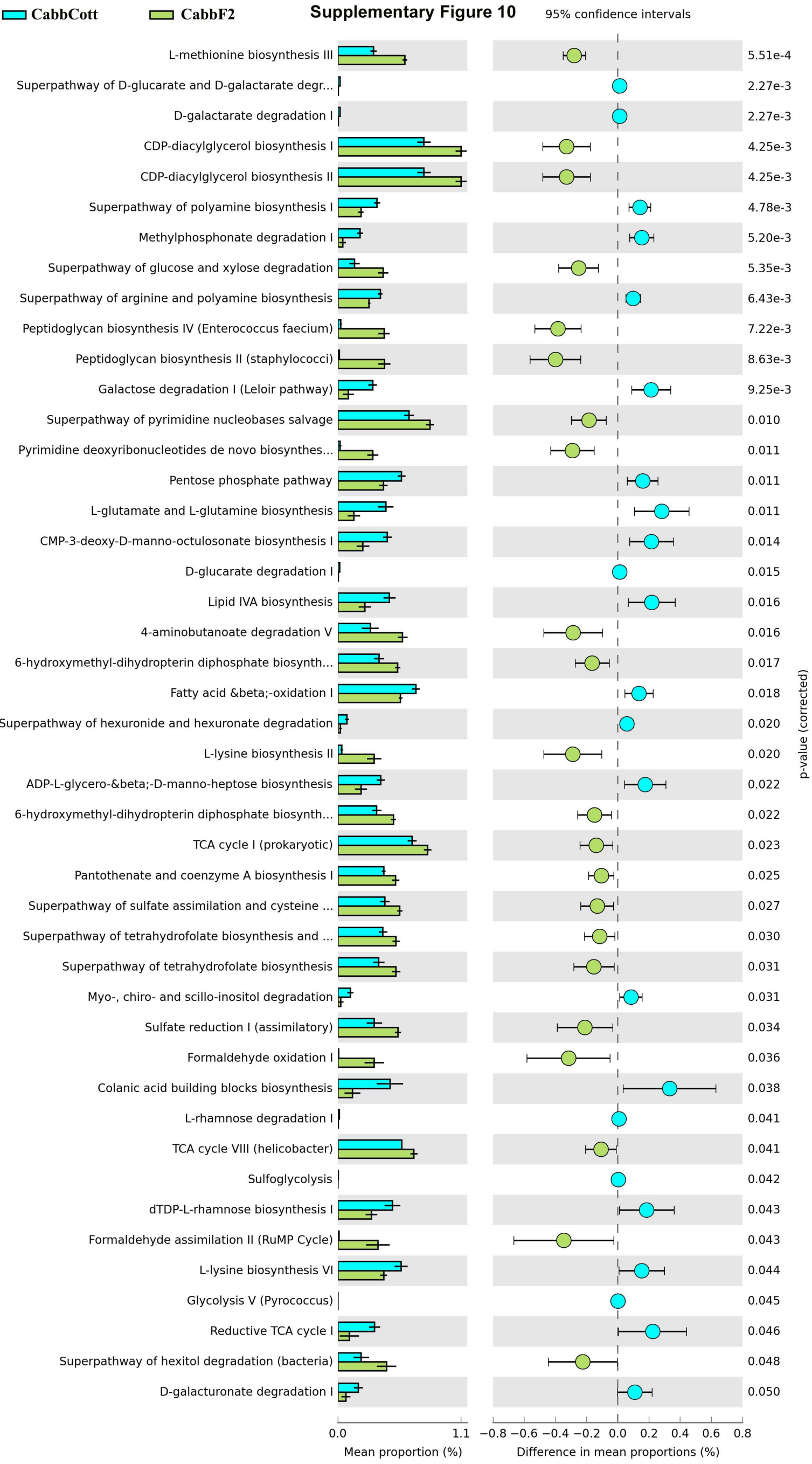

Supplement: Supplementary Figure 1 — Heatmap exhibiting the relative abundance of OTUs with an abundance cutoff of 5000 across all the feeding treatments. [file Data_Sheet_1.PDF]
