# Supplementary material for: Impact of transgenerational host switch on gut bacterial assemblage in generalist pest, Spodoptera littoralis (Lepidoptera: Noctuidae)
Source: Front Microbiol. 2023 Jul 13;14:1172601. doi: 10.3389/fmicb.2023.1172601 (PMC10374326; doi:10.3389/fmicb.2023.1172601)
Supplement: Supplementary Table 1 — Core gut bacteriome of S. littoralis larvae. Numbers in columns represent the average DESeq2 normalized read count for each feeding experiment. [file Data_Sheet_2.PDF]

Supplementary table 1. Core gut bacteriome of *S. littoralis* larvae. Numbers in columns represent the average DESeq2 normalized read count for each feeding experiment.

**OTUs**	**AD**	**CabbF1**	**CabbF2**	**CottF1**	**CottF2**	**CabbCott**	**CottCabb**
p_Actinobacteria_g_Actinomyces	3.60	0.27	0.18	0.00	2.49	14.95	57.97
p_Actinobacteria_g_Corynebacterium	60.89	121.78	174.24	168.39	102.74	81.03	101.09
p_Actinobacteria_g_Rhodococcus	3.91	1.09	28.87	3.90	0.00	2.79	0.00
p_Actinobacteria_g_Agromyces	15.07	0.27	4.95	22.31	13.12	13.82	0.00
p_Actinobacteria_g_Leucobacter	12.01	0.00	17.36	27.08	7.08	4.95	0.00
p_Actinobacteria_f_Microbacteriaceae_g_unclassified	95.53	23.47	0.00	12.68	44.45	5.69	13.64
p_Actinobacteria_g_Arthrobacter	20.30	0.00	67.00	206.80	42.70	27.07	0.00
p_Actinobacteria_g_Micrococcus	5.85	9.54	14.89	4.00	10.76	30.60	50.02
p_Actinobacteria_g_Actinoplanes	1666.70	0.54	5.73	21.36	0.00	32.39	8.68
p_Actinobacteria_g_Propionibacterium	36.28	46.62	55.59	80.70	45.30	55.25	56.49
p_Actinobacteria_g_Rubrobacter	3.09	19.37	27.24	11.47	21.41	7.07	11.31
p_Bacteroidetes_g_Prevotella	0.39	11.59	5.09	30.97	19.39	58.02	139.56
p_Bacteroidetes_g_Cloacibacterium	6.86	0.00	30.17	3.37	95.97	25.26	0.00
p_Bacteroidetes_g_Flavobacterium	53.15	48.46	173.13	42.02	21.06	14.54	107.70
p_Bacteroidetes_g_Chitinophaga	40.11	11.29	28.26	92.63	13.92	58.41	0.00
p_Firmicutes_g_Bacillus	18.94	61.56	35.19	25.92	7.46	68.46	123.89
p_Firmicutes_g_Brevibacillus	397.34	212.94	156.99	800.67	118.33	294.81	40.49
p_Firmicutes_g_Enterococcus	136.65	2762.70	203.39	906.67	1707.70	111.75	1817.60
p_Proteobacteria_g_Brevundimonas	0.00	3.40	0.00	14.50	12.93	17.99	20.25
p_Proteobacteria_g_Bosea	110.30	0.82	3.10	15.76	3.98	34.81	0.00
p_Proteobacteria_g_Bradyrhizobium	2014.80	461.92	616.73	2563.80	679.05	1428.70	103.38
p_Proteobacteria_g_unclassified	1.81	0.14	20.59	4.22	23.02	0.00	0.00
p_Proteobacteria_g_Methylobacterium	1096.80	426.57	404.93	1829.80	297.93	1066.10	176.12
p_Proteobacteria_g_Microvirga	53.66	29.19	11.81	25.10	0.00	6.34	0.00
p_Proteobacteria_g_Paracoccus	12.51	86.16	21.15	29.18	58.62	0.64	0.00
p_Proteobacteria_g_Sphingomonas	1189.90	1329.40	361.28	3149.00	421.44	1480.30	193.97
p_Proteobacteria_g_Cupriavidus	1280.30	401.77	933.98	5417.80	1128.80	1740.40	20720.00
p_Proteobacteria_g_Ralstonia	0.56	22.54	49.21	61.48	3911.60	199.90	83.84
p_Proteobacteria_g_Massilia	427.75	6.07	239.25	60.13	989.28	14.46	36.32
p_Proteobacteria_g_Enhydrobacter	0.00	0.27	12.58	43.89	73.52	34.24	0.00
p_Proteobacteria_f_Pseudomonadaceae_g_unclassified	0.00	0.00	33.98	0.16	10.70	14.48	0.41
